# Single‐cell landscape of the intrahepatic ecosystem in alcohol‐related liver disease

**DOI:** 10.1002/ctm2.70198

**Published:** 2025-01-20

**Authors:** Xiaofang Zhao, Senyan Wang, Qi Liu, Wenjuan Wei, Xiaoyan Sun, Hao Song, Jing Xu, Shuijun Zhang, Hongyang Wang, Jing Fu

**Affiliations:** ^1^ Translational Medicine Center The First Affiliated Hospital of Zhengzhou University Zhengzhou Henan China; ^2^ International Cooperation Laboratory on Signal Transduction, National Center for Liver Cancer, Ministry of Education Key Laboratory on Signaling Regulation and Targeting Therapy of Liver Cancer, Shanghai Key Laboratory of Hepatobiliary Tumor Biology Eastern Hepatobiliary Surgery Hospital, Second Military Medical University/NAVAL Medical University Shanghai China; ^3^ Department of Hepatobiliary and Pancreatic Surgery The First Affiliated Hospital of Zhengzhou University Zhengzhou Henan China

**Keywords:** ALB^+^KRT7^+^ epithelium, alcohol‐related liver disease, single‐cell RNA‐seq, Wnt/β‐catenin

## Abstract

Alcohol‐related liver disease (ALD) is a common chronic liver disease caused by long‐term excessive alcohol consumption and responsible for more than half of all liver‐related deaths worldwide. The molecular mechanisms associated with ALD were not fully understood. In this study, we performed single‐cell RNA sequencing on liver tissues obtained from ALD patients and healthy liver donors. We identified an ALB^+^KRT7^+^ epithelial population that expressed both hepatocyte and biliary markers significantly expanded in ALD livers. The ALB^+^KRT7^+^ epithelial cells were demonstrated to have stem cell properties and malignant transformation potentials. Moreover, ALB^+^KRT7^+^ epithelium‐derived ALD organoids promote the tumour growth by activating Wnt/β‐catenin signalling of liver cancer cells. Most importantly, blocking the Wnt protein secretion or knockdown the Wnt receptor suppressed the tumour promoting effect of ALD organoids. Our study provides important insights that Wnt signalling can be targeted in patients with advanced alcohol‐related cirrhosis to prevent malignant transformation. In addition, our results also uncovered the important alterations of nonparenchymal cells, especially macrophages and T/NK populations that responsible for active inflammation responses in alcohol‐related hepatitis and immunosuppressive microenvironment in advanced cirrhosis livers, which likely facilitated the malignant progression of ALD.

**Key points:**

- This study provides single‐cell landscape of human liver samples across different ALD stages.
- The ALB^+^ KRT7^+^ epithelium were enriched in ALD patients, and the function of this epithelial population varied significantly across ALD stages.
- ALB^+^KRT7^+^ epithelium from advanced alcohol‐related cirrhosis had malignant transformation potential and tumour promotion activity.
- The comprehensive changes of parenchymal and nonparenchymal cells in the ALD livers lay a hidden danger for the further malignant progression.

## Graphical abstract

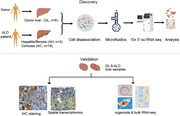

## 1 INTRODUCTION

Alcohol‐related liver disease (ALD) is a common chronic liver disease resulted by chronic excessive alcohol consumption and responsible for more than half of all liver‐related deaths worldwide.^1^, ^2^, ^3^ ALD progresses through a broad spectrum of diseases, including alcohol‐related fatty liver (simple steatosis), hepatitis, advanced fibrosis, and often progresses to cirrhosis and even liver cancer or liver failure.^4^, ^5^ As well known, cirrhosis is the premalignant stage of liver cancer, for which liver transplantation represents the only curative treatment, and a significant proportion of patients with alcohol‐related cirrhosis will progress to liver cancer.^1^ At present, alcohol‐related liver cancer accounts for about 30% of the global liver cancer incidence.^6^ However, the molecular mechanisms associated with human ALD progression were not fully understood.

The liver is the major organ for alcohol metabolism and the most important target organ for alcohol‐related damage. Previous studies have demonstrated that the toxic effects of alcohol and its intermediate metabolite cause a variety of biological processes, including oxidative stress, mitochondrial damage, lipogenesis, hepatocyte apoptosis and activation of hepatic stellate cells, etc.^7^, ^8^ All these factors are involved in the occurrence and progression of alcohol‐related cirrhosis. Nevertheless, the liver is a complex organ that contains numerous cell types with parenchymal cells and nonparenchymal cells, the exact cellular composition and the contributions of different cell type to the pathogenesis of ALD are widely unknown. Recent advancement in single‐cell RNA sequencing (scRNA‐seq) technology enabled us to identify heterogeneity of cell populations for human diseases. Recent ALD scRNA‐seq studies either performed scRNA‐seq on short‐term alcoholic liver injury mouse models or predicted cell type changes in ALD via gene deconvolution.^9^, ^10^ Of note, Guan et al. compared scRNA profiles between alcohol‐related cirrhosis (AC) and severe alcohol‐related hepatitis (sAH), and identified a neutrophils population that drive inexorable inflammation in sAH.^11^ However, these studies have largely focused on nonparenchymal cell populations rather than parenchymal cells. Unravelling the intricacies of both parenchymal and nonparenchymal cells are required to improve our understanding of the molecular mechanisms behind human ALD and and develop targeted therapeutic strategies.

In the present study, we systematically profiled and analysed single‐cell transcriptomes from 30 human liver samples across normal livers, alcohol‐related hepatitis/fibrosis and advanced alcohol‐related cirrhosis. Cellular composition, function, and crosstalk across cell types were depicted. Our study focused on the epithelial changes in ALD patients and identified an ALD‐enriched epithelial population‐ALB^+^ KRT7^+^ epithelium, that characterised by expressing both hepatocyte and biliary markers with stem cell characteristics and malignant transformation potential. Our analysis showed that ALB^+^KRT7^+^ epithelium in advanced alcohol‐related cirrhosis had increased Wnt activities, and inhibition of Wnt signalling can prevent the tumour promotion effect of ALB^+^ KRT7^+^ epithelium in organoid and murine models. Our data potentially shed light on the mechanism underlying alcohol associated liver disease progression, especially for alcohol‐related cirrhosis‐liver cancer transformation, which might guide the development of therapies to ALD patients.

## 2 MATERIALS AND METHODS

### 2.1 Human specimens

Human liver samples were collected at the First Affiliated Hospital of Zhengzhou University, Zhengzhou, China. The liver samples were processed for scRNA‐seq, histology, RNA isolation, or organoid generation. Details for patients were shown in Table S1. All patients’ diagnoses were histologically confirmed. This study was approved by the Ethical Committee of the First Affiliated Hospital of Zhengzhou University (2022‐KY‐0826‐001) and was conducted in accordance with the Declaration of Helsinki. Each participant had signed the informed consent.

### 2.2 Single‐cell RNA sequencing and analysis

Fresh liver tissues were surgically resected from patients and immersed in tissue storage solution (Miltenyi) and transported to the lab in a refrigerated container.

Liver tissues were dissociated for single‐cell suspensions following the standard manufacturer's protocols. The 10x Chromium Single cell 5′ Library (10x Genomics, V3 barcoding chemistry) were used to construct the sequencing libraries. Illumina Hiseq X Ten sequencer was utilised to analyse purified libraries.

Cell Ranger (Version 6.1.2) Pipeline coupled with human reference version GRCh38‐2020‐A was used to generate raw gene expression matrices. Then the filtered gene expression matrices were analysed by using R software (Version 4.1.2) and the Seurat package (Version 4.1.1). The Scrublet (Version 0.2.1) was used to remove the potential doublets and low quality cells (n_genes > 200, n_counts > 500, percent_mito < 10%). Detailed analysis method were described in Supplementary Methods.

### 2.3 Organoid culture

Fresh liver specimen was minced and incubated in digestion solution (2.5 mg/mL Collagenase IV, Roche) for 30 to 60 min at 37°C. The digestion was stopped by adding DMEM with 10% FBS, then the suspension was filtered through a 100 µm cell strainer and centrifugated at 1000 rpm for 5 min. The pellet was washed with cold Advanced DMEM/F12 (GIBCO, USA), and mixed with matrigel (Corning, USA). Cells were seeded and cultured in 6‐well suspension plate (10 000–20 000 cells per well). The organoid culture medium for normal and ALD samples consisted of Advanced DMEM/F12 medium, 1:50 B27 supplement, 1.25 mM N‐acetyl‐l‐cysteine, 250 ng/mL Rspo‐1, 100 ng/mL Wnt3a, 100 ng/mL Noggin, 50 ng/mL EGF, 100 ng/mL FGF10, 25 ng/mL recombinant human HGF, 10 mM Y27632, 10 mM nicotinamide, 5 µM A8301, 10 µM forskolin, 1:500 Primocin, 1% penicillin/ streptomycin, 1% Glutamax, and 1% HEPES.

The organoid line L010 and AL008 (5×10^5^ cells per well) were seeded in 6‐well dishes, and cultured in liver organoid culture medium for 3 days. To block Wnt secretion, L010 and AL008 organoids were treated with 2 µM IWP‐2 (Selleck, China) or 1 µM LGK‐974 (Selleck, China). Control groups were treated with equality of DMSO. After 48 h, media was collected for conditioned media experiments.

### 2.4 HCC spheroids cultured with liver organoid supernatant

A total of 3000 HCCLM3 cells were seeded in a droplet of 50 µL containing advanced DMEM/F12‐10% FBS media in suspension plate for 10 days to form HCC spheroids. Then, HCC spheroids were transferred in droplets of 50 µL of organoid conditioned media (L010 SN or AL008 SN). Pictures of the HCC spheroids were taken at day 0 and day 7, and spheroid growth was measured with Image View software.

### 2.5 Construction of si‐FZD5 HCC cell line

The siFZD5‐HCCLM3 cell line was constructed using the lentiviral vector GV112 (hU6‐MCS‐CMV‐Puromycin, siFZD5 sequence: CGGCATCTTCACGCTGCTCTA, purchased from Genechem, China). HCCLM3 were seeded in 6‐well plates (5×10^5^ cells per well) and infected with siFZD5 lentiviral vector according to the manufacturer's instructions.

### 2.6 Tumour xenograft

Liver orthotopic xenograft assays were conducted with 6‐week‐old male NSG mice (The Jackson Laboratory). The mice were randomly divided into three groups: HCCLM3 only, HCCLM3+L010 organoids and HCCLM3+AL008 organoids (*n* = 6). L010 or AL008 organoids were mixed with HCCLM3 cells (2×10^6^ per mice) in a 1:10 ratio, and indicated cells were injected under liver capsule to generate orthotopic implantation model. Mice in the control group (HCCLM3 only) were only implanted with HCCLM3 cells (2×10^6^ per mice).

For the inhibitor treatment, L010 and AL008 organoids were treated with 1 µM LGK‐974 for 48 h before transplanted to mice. Then inhibitor‐treated L010 or AL008 organoids were mixed with HCCLM3 cells (2×10^6^ per mice) in a 1:10 ratio and implanted in to mouse livers. Mice in control group were only implanted with HCCLM3 cells (2×10^6^ per mice). A week after transplantation, LGK‐974, dissolved in vehicle (5% DMSO and 95% corn oil), was injected intraperitoneally to all mice in the three groups at a dose of 3 mg/kg every day for 14 days. Then the mice were sacrificed and liver samples were excised for further analysis. Tumour diameter was measured by using a calliper, and tumour volume was calculated following: (width)^2^ ×length/2. All animals were housed in a specific pathogen‐free environment with free access to food and water and a 12‐h dark/light cycle. All animal experiments were approved by the Ethical Committee of the First Affiliated Hospital of Zhengzhou University and cared for in accordance with the National Institutes of Health Guide for Laboratory Animals.

### 2.7 Histology and staining

The paraffin embedding of liver tissues and organoids were performed following standard protocols. The embedded samples were cut into 5 µm and prepared for H&E and immunohistological (IHC) or immunofluorescence (IF) staining according to a standard protocol. For immunostaining, primary antibodies against ADH4 (ab137077, Abcam, 1:1000), Perilipin2 (15294‐1, Proteintech, 1:500), FXYD2 (11198‐1, Proteintech, 1:200), ALB (ab192603, Abcam, 1:300 for IHC; ab207327, Abcam, 1:500 for IF), CK7 (NBP2‐44814, Novus Biologicals, 1:50), Flag (14793S, Cell Signaling Technology, 1:500), β‐catenin (17565‐1, Proteintech, 1:200), AQP1 (20333‐1, Proteintech, 1:2000), CXCL6 (SC‐377026, Santa Cruz, 1:100), CCN1 (39382, Cell Signaling Technology, 1:100), WNT7A (10605‐1, Proteintech, 1:500), CD68 (GB115723, Servicebio, China), and FN1 (GB5091, Servicebio, China) were used.

### 2.8 Bulk RNA isolation and sequencing

Total RNA isolation was performed using RNeasy mini kit (Qiagen, Germany). The TruSeq Stranded Total RNA Sample Preparation kit (Illumina, USA) was applied to construction Strand‐specific libraries. The construction and sequencing of RNA libraries were performed at OE Biotechnology Corporation, China.

### 2.9 Statistics

Data analysis was performed by using the SPSS software (version 16; SPSS). Each experiment was in triplicate at least and values were presented as mean ± SD. Statistic differences were calculated using Student's *t*‐test and Wilcoxon test. Values of *p* < .05 were considered statistically significant.

## 3 RESULTS

### 3.1 Single‐cell transcriptomic landscape of human alcohol‐related livers

**FIGURE 1:**
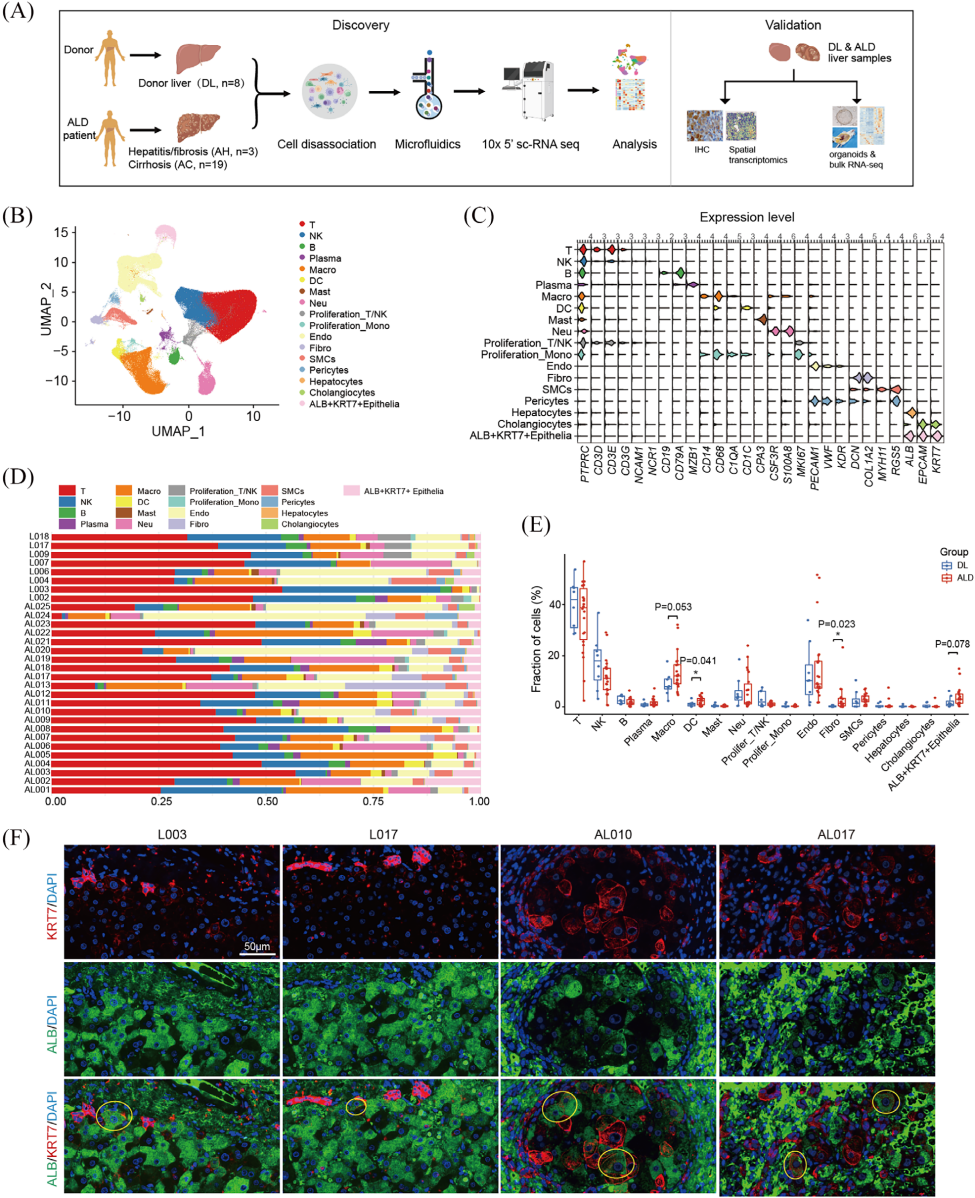
Single‐cell Atlas of patient‐derived alcoholic and normal liver tissues. (A) Graphic overview of study design for sample collection, processing, sequencing, data analysis, and external validation. (B) UMAP plots visualising 17 colour‐coded major cell types. (C) Violin plots showing expression levels of known marker genes for each cell type. (D) Histogram indicating the proportion of cells in liver tissue of each analysed patient. Source data are provided as Table S1. (E) Box plots showing the proportions of major cell types in donor liver (DL) and alcohol‐related liver disease (ALD) group. (F) Representative immunofluorescence images of ALB and KRT7 in DL (L003 and L017) and alcohol‐related cirrhosis (AL010 and AL017) liver tissue sections. Yellow circles indicate the distribution of ALB+KRT7+ epithelial cells (EPCs). Scale bar, 50 µm. *p* values in E were calculated by two‐tailed Student's *t*‐test. *p < .05.

To elucidate the cellular composition and heterogeneity of human ALD livers, we performed 5′ scRNA‐seq on liver samples from 8 healthy donors (donor liver, DL), 3 patients with alcohol‐related hepatitis or fibrosis (AH) and 19 patients with advanced alcohol‐related cirrhosis (AC) (Figure 1A). Detailed clinical and pathological information is provided in Table S1.

After quality control, filtering and doublet removal, a total of 288 401 cells were included. Based on top differentially expressed genes (DEGs), the cells were classified into 17 major cell types by graph‐based uniform manifold approximation and projection (UMAP), including T cells, natural killer (NK) cells, B cells, plasma cells, macrophages, dendrite cells, mast cells, neutrophils, cycling cells, endothelial cells, fibroblasts, smooth muscle cells, pericytes and epithelial cells (Figures 1B and C and S1A and Table S2). Intriguingly, we noticed that epithelial cells formed 3 distinct subtypes, including cholangiocytes (marked with *KRT7*, *KRT19*, *MUC5B*), hepatocytes (marked with *ALB, TTR*), and ALB^+^KRT7^+^ cells, which simultaneously expressed hepatocyte markers and cholangiocyte markers, exhibiting an intermediate state (Figures 1C and S1A). Moreover, ALB^+^KRT7^+^ epithelial cells expressed high levels of liver progenitor cell markers, for example *EPCAM, SOX9* and *CD24* (Figures 1C and S1A). All major cell types were presented in both normal and ALD tissues (Figure 1D). Overall, the fractions of macrophages (*p* = .053), DCs (*p* = .041), fibroblasts (*p* = .023) and ALB^+^KRT7^+^ EPCs (*p* = .078) increased in ALD livers compared with control donor livers (Figures 1E and S1B). Further analysis across different disease stages showed that macrophages expanded in both AH and AC samples, indicating possible roles in early pathogenesis of ALD (Figure S1C). In addition, ALB^+^KRT7^+^ EPCs showed evident increase since AH stage (Figure S1C). The immune staining of ALB and KRT7 in liver sections suggested the increased ALB^+^KRT7^+^ EPCs in ALD samples (Figure 1F).

### 3.2 Characteristics of hepatocytes

ALD is a metabolic liver disease arising from the alcohol‐mediated parenchymal cellular injury; here we paid close attention to parenchymal cell alterations. By comparing the gene expressions of hepatocytes, we observed prominent differences between hepatocytes of ALD and DL. In comparison with DL livers, hepatocytes from ALD livers showed upregulation of, for example, progenitor markers such as *EPCAM, CD24* and *MYC*. While, genes related with liver metabolic functions, such as *HMGCS1, ALDH2* and *ADH4*, were downregulated (Figure S2A). Gene Set Enrichment Analysis (GSEA) revealed that epithelial mesenchymal transition (EMT) and extracellular matrix organisation signalling were enriched in ALD hepatocytes (Figure S2B).

**FIGURE 2:**
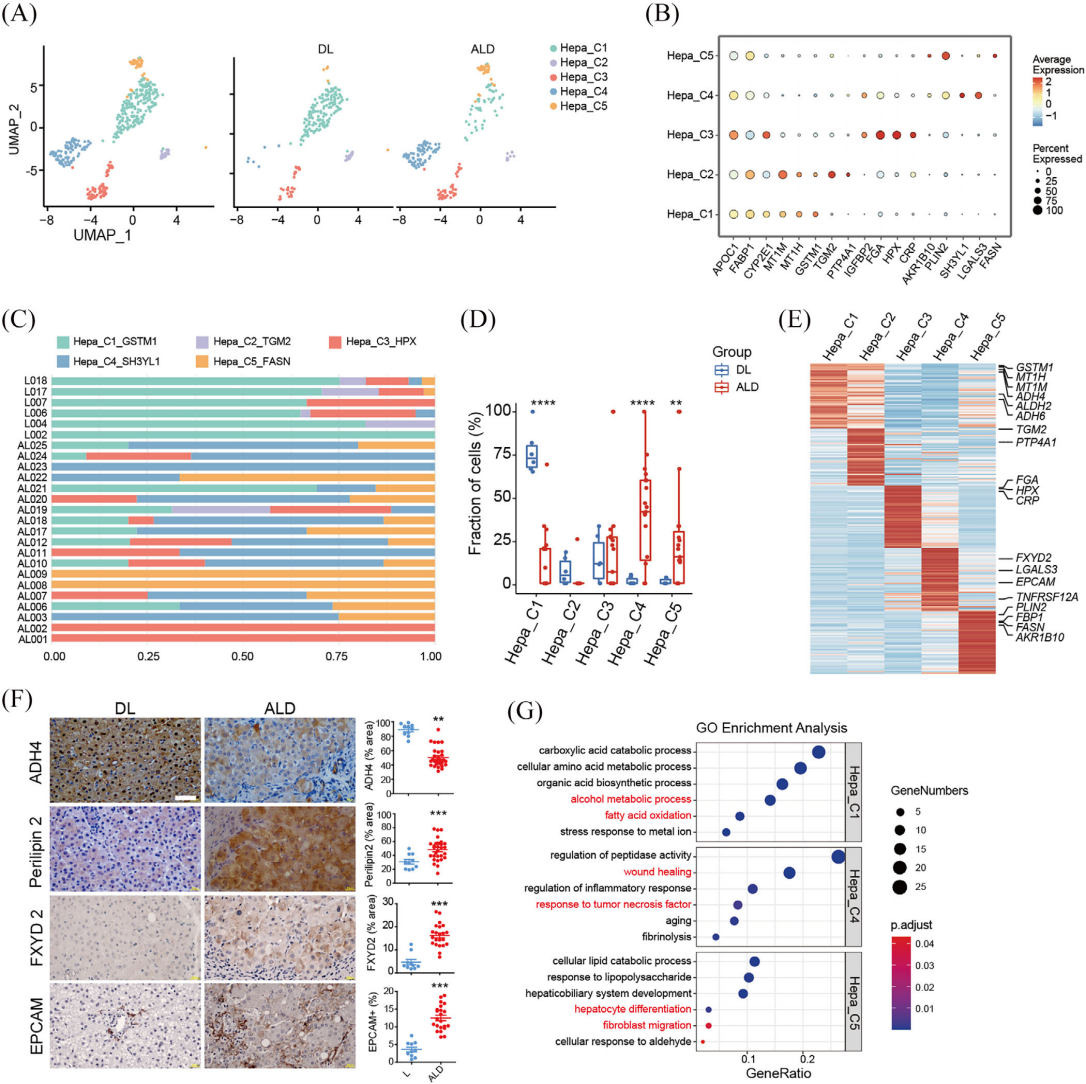
Characteristics of hepatocytes (A) UMAP plots of hepatocytes subsets in all samples (left) and in different groups (right). (B) Bubble plot showing expression levels of marker genes across hepatocyte subsets. (C, D) Histogram (C) and box plots (D) showing the proportions of hepatocyte subsets in samples from DL and ALD group. (E) Heatmap indicating the expression of top‐ranking DEGs in hepatocyte subclusters. (F) Representative IHC staining images of ADH4, Perilipin2, FXYD2 and EPCAM in DL and ALD samples (left). Scale bars, 50 µm. Boxplot illustrating the fraction of positive cells, in normal (DL, *n* = 10) and ALD (*n* = 30) samples based on IHC results (right). (G) Dot plots showing typically significantly enriched GO terms across hepatocyte subsets. *p* Values in D and F were calculated by two‐tailed Student's *t*‐test. ** *p* < .01; ***p < .001, ****p < .0001.

To understand detailed alterations, all hepatocytes were reclustered into five subsets, Hepa_C1 to C5 (Figure 2A and B). The distribution of these subsets were remarkably different between DL and ALD livers, with Hepa_C1 significantly decreased in ALD livers, while Hepa_C4 and C5 specifically expanded in ALD livers (Figure 2C and D). Accounting for the main hepatocyte population in DL livers, Hepa_C1 expressed high levels of alcohol metabolic genes, such as *ADH4*, *ADH6* and *ALDH2*, and the proportion of ADH4^+^ hepatocytes was consistently reduced in ALD liver sections (Figure 2E and F).^12^ Gene oncology (GO) analysis confirmed the enrichment of pathways of alcohol metabolic process in Hepa_C1 (Figure 2G). DEG analysis showed that Hepa_C4 and C5 expressed high levels of Perilipin 2 (PLIN2), a molecule that promotes the formation and stabilisation of the intracellular lipid droplets. Immunohistochemical staining validated the increase of PLIN2^+^ hepatocytes in ALD liver sections, which was consistent with previous study (Figure 2E and F).^10^ Intriguingly, we found that Hepa_C4 and C5 also expressed increased EPCAM and cholangiocyte marker FXYD2, which were validated by immune staining (Figure 2E and F). GO analysis showed the wound healing pathways and TNFα signalling were enriched in the Hepa_C4, and hepatocyte differentiation were enriched in C5 subset (Figure 2F). These findings are consistent with previous reports about the hepatocyte dedifferentiation in response of chronic injuries.^13^, ^14^

### 3.3 Stem cell characteristics of ALB^+^KRT7^+^ epithelium

**FIGURE 3:**
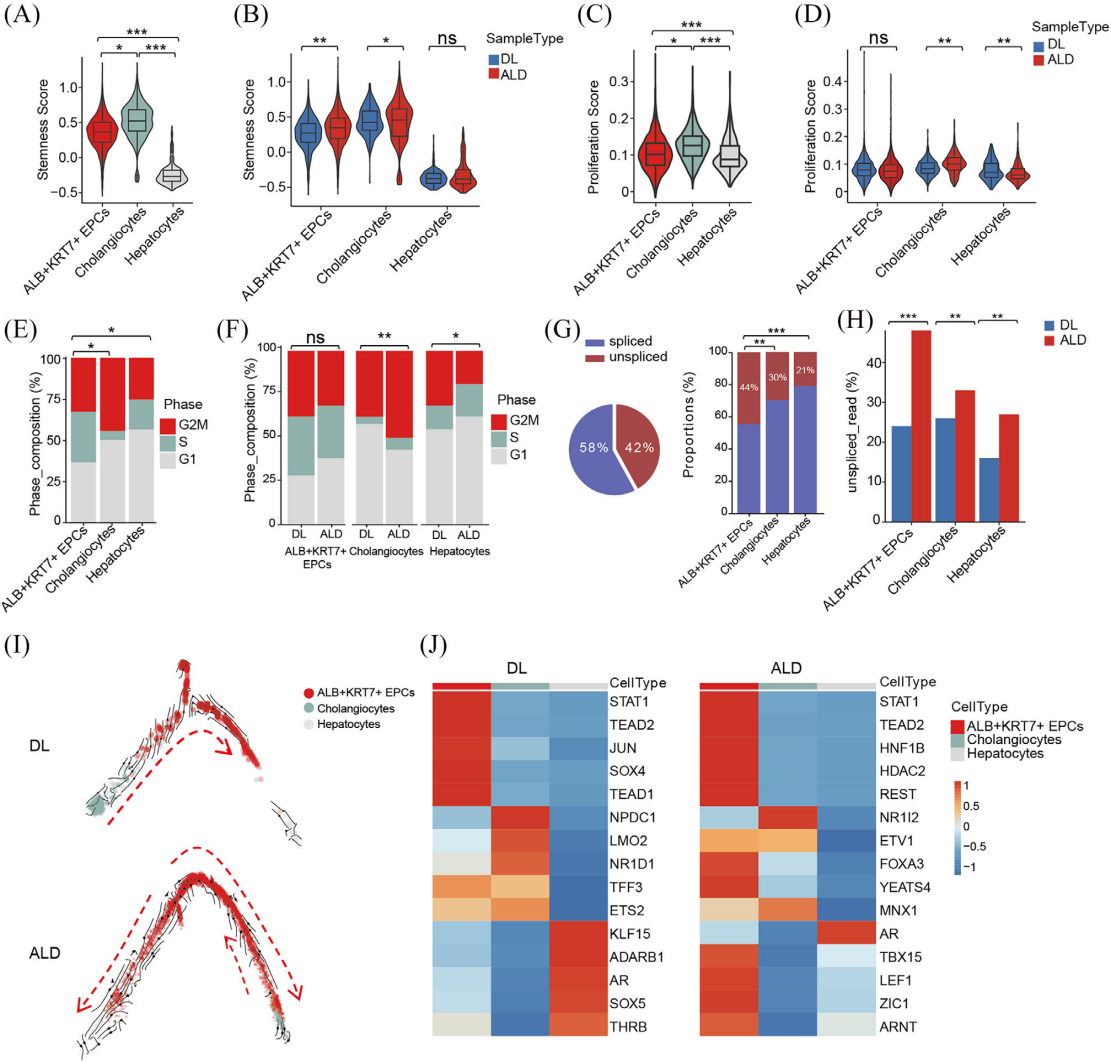
Stem cell Characteristics of ALB^+^KRT7^+^ Epithelium. **(A, B)** Violin plot comparing stemness scores across epithelial populations **(A)** and between DL and ALD groups **(B)**. The **s**temness scores were calculated according to the expression levels of of stem cell marker genes, including *EPCAM, SOX9, CD24, CD44, OCT4, SOX2, FOXL1,TROP2, LGR5, OV6, A6* and *CD133*, in each epithelial populations. **(C, D)** Violin plot displaying proliferation scores for each epithelial cell type **(C)** and between DL and ALD groups **(D)**. **(E, F)** Histogram indicating the proportion of cell cycle distribution of each epithelial cell type **(E)** and between DL and ALD groups **(F)**. **(G)** Pie chart showing the proportions of spliced or unspliced RNA in all epithelium and histogram showing the proportions of unspliced RNA in each epithelial cell type. **(H)** Histogram comparing the proportion of unspliced RNA in epithelial cells between DL and ALD group. **(I)** RNA velocity analysis showing the transition potential among ALB^+^KRT7^+^ EPCs, cholangiocytes and hepatocytes from DL and ALD group, respectively. **(J)** Heatmap showing transcription factor activity of epithelial subsets among DL and ALD group. *p* Values in **A** to **H** were calculated by two‐sided unpaired Wilcoxon test. *p < .05, **p < .01; ***p < .001, ****p < .0001, ns, not significant.

Studies have demonstrated the existence of stem cell or progenitor populations in adult livers, and those cells have long been suggested involved in liver regeneration.^15^, ^16^ However, the identity of these populations in ALD remains elusive. Here, we speculated the ALB^+^KRT7^+^ epithelium, expressing high levels of *EPCAM* and *SOX9*, is the stem cell subset. To further evaluate the stemness of epithelial populations, we used stemness score defined by the expression of reported stem cell marker genes, such as *EPCAM, SOX9, CD24*, and *CD44*.^17^, ^18^, ^19^ The ALB^+^KRT7^+^ EPCs and cholangiocytes showed dominant stem cell phenotype (Figure 3A). Moreover, ALB^+^KRT7^+^ EPCs from ALD group exhibited higher stemness scores than those from control donor livers (Figure 3B). To further character the ALB^+^KRT7^+^ epithelium, we checked the cell proliferation capacities. Gene set score showed higher proliferation score in ALB^+^KRT7^+^ EPCs than mature hepatocytes (Figure 3C). Consistently, ALB^+^KRT7^+^ EPCs had significantly higher proportions of S and G2M phase cells, especially dramatically increased S phase cells, in comparison with mature hepatocytes and cholangiocytes, indicating active cell proliferation potential (Figure 3E). In addition, cholangiocytes from ALD samples had more S and G2M proportions than those from DL group (Figure 3F), implying increased cholangiocyte proliferation, which was in agreement with previous described ductular reaction in chronic liver disease.

We next explored the dynamic trajectories of epithelial cells using RNA velocity.^20^ RNA splicing state analysis revealed that unspliced RNA were more abundant in ALB^+^KRT7^+^ EPCs than hepatocytes and cholangiocytes, further confirming their progenitor properties (Figure 3G). Interestingly, the proportions of unspliced RNA in all epithelial populations were much higher in ALD compared to DL livers implying increased transcription rate in ALD epithelium (Figure 3H). The pseudotemporal trajectory in DL showed that cholangiocytes were at the beginning of the trajectory path and transformed to ALB^+^KRT7^+^ EPCs (Figure 3I). Whereas nearly no lineage connection between cholangiocytes/ALB^+^KRT7^+^ EPCs and hepatocytes, indicating that ALB^+^KRT7^+^ EPCs originated from cholangiocytes, and remained quiescent in normal condition. Of note, in the ALD group, there existed more complex trajectories. Besides the transformation path from cholangiocytes to ALB^+^KRT7^+^ EPCs, there was a bidirectional transformation originating from ALB^+^KRT7^+^ EPCs. The ALB^+^KRT7^+^ EPCs transformed to both hepatocytes and cholangiocytes, implying a bipotent progenitor capacity (Figure 3I). In addition, SCENIC analysis showed that ALB^+^KRT7^+^ EPCs from ALD livers tuned up more regulons that were reported to promote liver progenitors expansion, such as *HNF1β* and *FOXA3*, in comparison with those from DL group (Figure 3J).^21^

### 3.4 Malignant transformation potency of ALB^+^KRT7^+^ EPC in alcohol‐related cirrhosis

**FIGURE 4:**
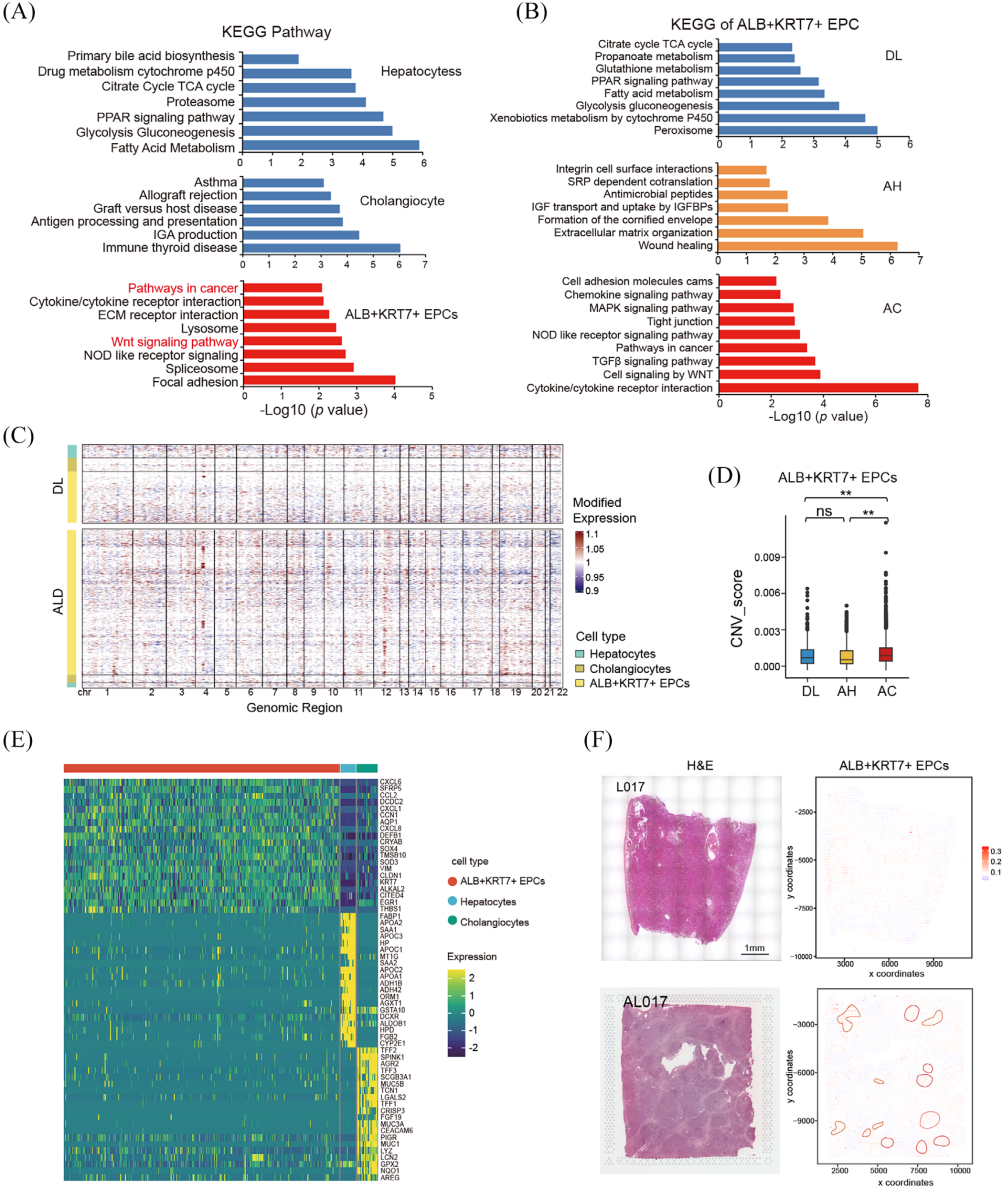
Malignant transformation potency of ALB+KRT7+ EPC in Alcoholic cirrhosis (A, B) Bar chart showing the enrichment of specific pathways in each epithelial cell type (A) and in ALB+KRT7+ EPC from DL, AH (alcohol‐related hepatitis) and AC (alcohol‐related cirrhosis) group (B), based on KEGG pathway analysis. (C, D) The landscape of inferred large scale CNVs (C) and CNV scores (D) for epithelial cells among samples from each group. (E) Heatmap comparing the specific signature genes in ALB+KRT7+ EPC, cholangiocytes and hepatocytes. (F) H&E staining and spatial feature plots of signature score of ALB+KRT7+ EPCs in tissue sections of L017 and AL017. Scale bar, 1 mm. *p* Values in D were calculated by two‐sided unpaired Wilcoxon test. **p < .01, ns, not significant.

To gain functional insight of the ALB^+^KRT7^+^ EPCs, we performed DEG and pathway analysis. Strikingly, KEGG analysis indicated that cancer‐related signal pathways were enriched in ALB^+^KRT7^+^ EPCs, such as Wnt signalling (Figure 4A). This led us to speculate that the ALB^+^KRT7^+^ EPCs involved in the malignant transformation of alcohol‐related cirrhosis. We next compared the pathway enrichment of ALB^+^KRT7^+^ EPCs across different ALD stages. The results showed that the hepatic function signals (e.g., glucose, fatty acid and xenobiotics metabolism pathways) were enriched in DL group; wound healing and ECM organisation signals were enriched in AH group; whereas Wnt signalling, cell proliferation and immune response pathways (e.g., chemokine and cytokine signalling) were enriched in AC group (Figure 4B). Moreover, Hallmark gene set for cancer‐related pathways (e.g., EMT, IL6 and TNFα signals) were exclusively enriched in ALB^+^KRT7^+^ EPCs from AC samples (Figure S3A). Interestingly, GO analysis suggested that pathway associated with cell morphogenesis and migration (e.g., ameboidal‐type cell migration, intermediate filament organisation, axon development, neuron projection extension) were obviously upregulated in ALB^+^KRT7^+^ EPCs from AC group, suggesting a migratory cell phenotype (Figure S3B). Given that malignant cells harbour enriched copy‐number variations (CNVs), we inferred large‐scale chromosomal CNVs based on RNA transcriptomes. The results showed that ALB^+^KRT7^+^ EPCs from AC samples exhibited obviously increased CNV compared with DL and AH groups (Figure 4C and D), indicating possible malignant phenotype. Moreover, by comparing gene expression profiles among ALB^+^KRT7^+^ EPCs, cholangiocytes and hepatocytes, we identified DEGs that specifically differentially expressed in ALB^+^KRT7^+^ EPCs relative to the other epithelial populations. Intriguingly, we found that ALB^+^KRT7^+^ EPCs expressed robustly high levels of chemokines, including CCL2, CXCL6, CXCL8 and CXCL1, indicating immunomodulating properties (Figure 4E). Moreover, CCN1 (also called CYR61), a YAP target gene, were significantly upregulated in ALB^+^KRT7^+^ population (Figure 4E), implicating increased YAP activity. Among these top upregulated genes, CXCL6, CCN1 and AQP1 were validated in AC liver tissue microarray (*n* = 45) as well as in DL (*n* = 12) and AH (*n* = 4) livers by IHC staining. The results showed that CXCL6^+^, AQP1^+^ or CCN1^high^ cells were absent or few in the donor livers, and gradually increased in ALD stages (Figure S3B and C). To investigate the spatial distribution of the ALB^+^KRT7^+^ EPCs, DL sample L017 and AC sample AL017 were subjected to spatial transcriptomics. Top expressed genes in ALB^+^KRT7^+^ EPCs were used to predict the spatial distribution of ALB^+^KRT7^+^ EPCs. The results showed that in L017, ALB^+^KRT7^+^ EPCs located near the bile duct, whereas in AL017, ALB^+^KRT7^+^ EPCs were more abundant and distributed in both edge and internal of pseudolobule (Figure 4F).

### 3.5 ALD Liver organoids derived from ALB^+^KRT7^+^ EPCs promote liver tumour growth

**FIGURE 5:**
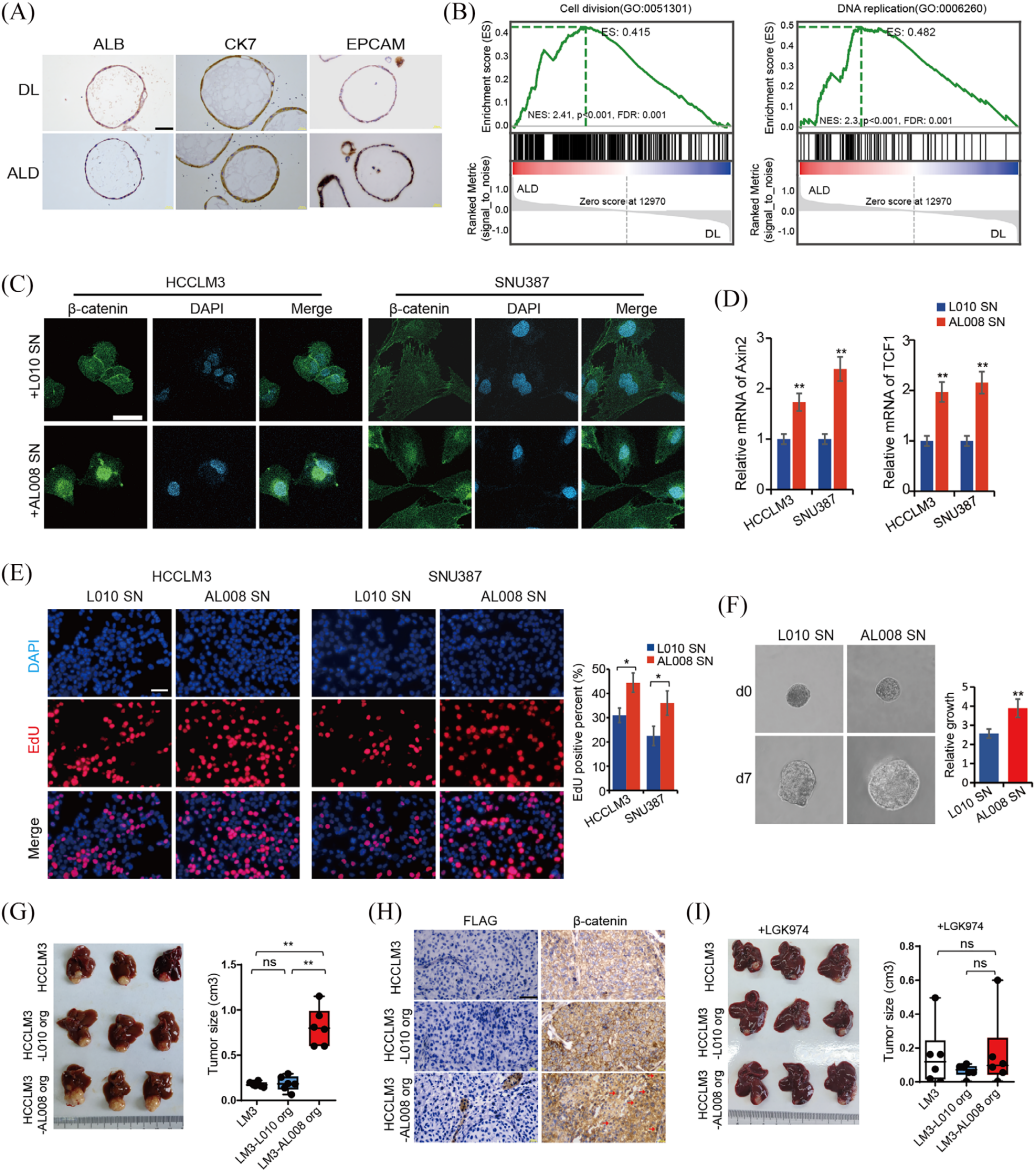
ALB+KRT7+ EPCs derived ALD liver organoids promote liver tumour growth. (A) Representative IHC staining images of ALB, KRT7 and EPCAM in organoids generated from DL and ALD livers. Scale bars, 50 µm. (B) Heatmap showing top‐ranking DEGs for organoids of DL and ALD groups, based on bulk RNA‐seq data. (C) Representative IF staining images of β‐catenin in HCCLM3 and SNU387 cells treated with supernatants (SN) of L010 organoids or AL008 organoids for 48 h. Scale bars, 50 µm. (D) Bar chart showing the Axin2 and TCF1 mRNA expression levels in HCCLM3 and SNU387 cells treated with L010 SN or AL008 SN for 48 h, compared by real time PCR. (E) Representative EdU staining images of HCCLM3 and SNU387 cells treated with L010 SN or AL008 SN for 48 h. Scale bars, 50 µm. Boxplot illustrating the fraction of EdU positive cells. (F) Representative HCCLM3 spheroid images cultured with L010 SN or AL008 SN. Boxplot comparing the growth ratios. (G) Representative liver tissue images and tumour size of NSG mice orthotopically transplanted with HCCLM3 without or with L010 organoids or with AL008 organoids (*n* = 6). (H) Representative IHC staining images of FLAG and β‐catenin in tumour sections of each group. Arrows indicate β‐catenin nuclear localisation. Scale bars, 50 µm. (I) Representative liver tissue images and tumour size comparison of LGK‐974‐treated NSG mice, orthotopically transplanted with indicated cells (*n* = 6). Data in G and I presented as the mean  ±  SD, *p* values were calculated by two‐sided unpaired Wilcoxon test. *p < .05, **p < .01, ns, not significant.

Moreover, we generated organoids from both DL and ALD liver tissues. It has been demonstrated that liver organoids were originated from stem cell populations, therefore, we speculate that these organoids were originated from ALB^+^KRT7^+^ EPCs. To validate this issue, we checked expression levels of ALB and KRT7 in these organoids. Immunohistochemical staining showed that organoid cells expressed both ALB and KRT7, as well as EPCAM, indicating ALB^+^KRT7^+^ EPCs phenotype (Figure 5A). Bulk RNA sequencing analysis showed that 223 genes were upregulated and 518 genes were downregulated (*p* < .05, Fold change ≥ 2) in ALD organoids compared to DL controls (Figure S4A). Among the DEGs, we noticed that Wnt negative regulators DKK3 and SFRP5 were significantly downregulated in ALD organoids, while the Wnt ligand protein Wnt7a remarkably upregulated (Figure S4A), indicating active Wnt signal in ALD organoids. Then the expression levels of Wnt7a were also determined in DL and ALD liver tissues, the IHC results suggested Wnt7a expression increased from DL to AH and AC livers in a progressive manner (Figure S4B). Furthermore, we observed that the Wnt7a expression levels in AC samples were significantly positively correlated with CXCL6^+^ and CCN1^high^ cell abundance (Figure S4C), implying associations between Wnt7a expression and ALB^+^KRT7^+^ EPCs cell expansion in ALD. Moreover, GSEA analysis revealed that cell division and DNA replication signals were significantly enriched in ALD organoids (Figure 5B). Additionally, stem cell development, angiogenesis and myoblast proliferation signals, which were highly correlated with tumourigenesis, were also upregulated in ALD organoids (Figure S4D). These together implicated possible tumour initiation or promotion roles of ALB^+^KRT7^+^ EPCs‐derived ALD organoids.

Given the vital role of the Wnt in HCC and the increased Wnt activity in ALB^+^KRT7^+^ EPCs‐derived ALD organoids, we next evaluated potential effects of ALD organoids on HCC growth. According to the expression levels of Wnt7a in organoid lines, AL008, with the highest Wnt7a expression, were selected for subsequent experiments, and donor liver organoid L010 was used as control (Figure S4C). To evaluate whether the ALD organoids affect HCC growth through paracrine Wnt‐dependent signalling, the HCC cell line HCCLM3 and SNU387 were incubated with supernatants from organoid AL008 or L010, respectively. Increased β‐catenin nuclear localisation, as well as increased mRNA levels of downstream effector Axin2 and TCF1 in AL008 supernatant‐treated HCC cells suggested increased Wnt/β‐catenin activity (Figure 5C and D). More importantly, the EdU and spheroid experiments demonstrated that HCC cells treated with AL008 supernatants exhibited increased cell proliferation and accelerated growth rate than those treated with L010 supernatant (Figure 5E and F). Then we blocked Wnt ligand secretion (with IWP‐2 or LGK‐974) in both organoids and observed that the growth‐promoting effect of AL008 organoid supernatant was abolished by IWP‐2 or LGK‐974 treatment (Figure S5A and B), implicating the effects of ALD organoids on HCC growth were dependent on Wnt secretion. Further evidence was obtained from experiments where we knockdown the Wnt receptor‐FZD5 by siRNA in HCCLM3 cells and observed that FZD5 silence abrogated the growth differences between AL008 and L010 organoid supernatant‐treated HCC cells (Figure S5C and D). Furthermore, liver orthotopic implantation model was applied to determine the in vivo effect of ALB^+^KRT7^+^ EPCs‐derived organoids on HCC growth. The results showed that both normal and ALD organoids were not able to form tumour in vivo. While, when implanted together with HCCLM3, increased tumour growth was observed in the ALD groups (Figure 5G). Immunohistochemical staining of Flag suggested the survival of ALD organoids in orthotopic tumour tissues, while control organoids were not detected in tumours (Figure 5H). In accordance with the in vitro findings, increased nuclear localisation of β‐catenin were observed in tumours of ALD group (Figure 5H). Furthermore, LGK‐974 treatment abrogated ALD organoids’ growth promotion effect on HCCLM3 tumours in vivo (Figure 5I). The immunostaining showed that although ALD organoids survived in HCCLM3 tumour tissues, evidenced by Flag expression, the promotion of nuclear β‐catenin by ALD organoids was abolished by LGK‐974 (Figure S5E). Collectively, our findings strongly suggested that ALB^+^KRT7^+^ EPCs originated organoids promoted tumour growth via Wnt/β‐catenin signalling.

Next, to investigate clinical relevance of ALB^+^KRT7^+^ epithelial cells in HCC promotion, we queried the TCGA database, by screening clinical information of the HCC samples from the LIHC cohort, we obtained 65 alcohol‐related HCC samples. with 45 samples survived more than one month. The expression levels of a gene set comprising top 20 DEGs in ALB^+^KRT7^+^ EPCs were scored in the alcohol‐related HCC samples. According to the ALB^+^KRT7^+^ EPCs scores, the 45 patients were divided into high‐ALB^+^KRT7^+^ EPCs and low‐ALB^+^KRT7^+^ EPC groups, survival analysis suggested that patients from high‐ALB^+^KRT7^+^ EPCs group had significant worse prognosis (Figure S6A). Furthermore, immune cell infiltration were compared between the low‐ and high‐ALB^+^KRT7^+^ EPCs group. Notably, we observed increased infiltration levels of Tregs, a pivotal immunosuppressive CD4^+^T cell that promote tumour progression, in high‐ALB^+^KRT7^+^ EPCs group (Figure S6B). These clinical findings further implied the tumour promotion effect of the ALB^+^KRT7^+^ epithelial cells in alcohol‐related HCC.

### 3.6 Characteristics of immune cells

**FIGURE 6:**
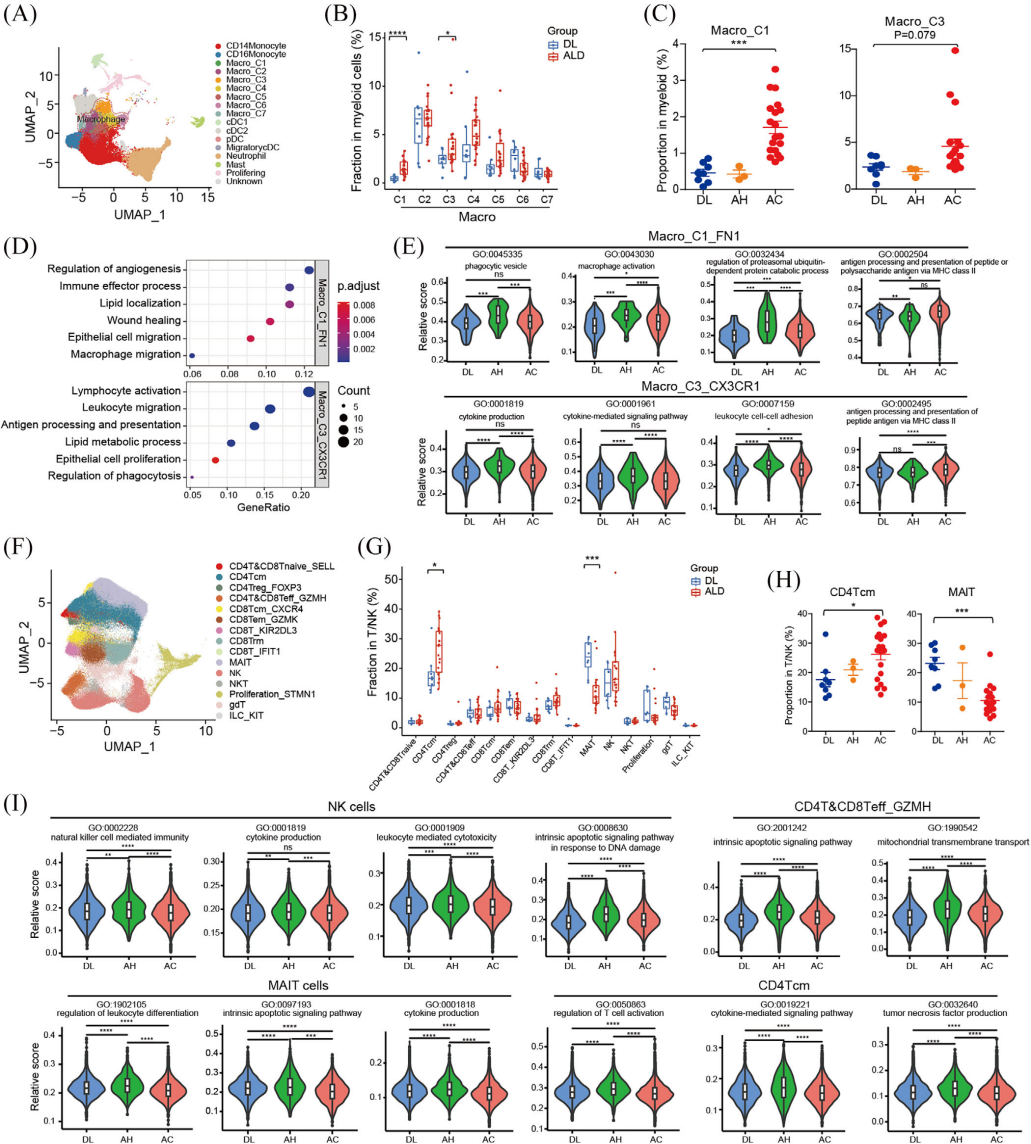
Characteristics of immune cells. (A) UMAP plots of myeloid subsets. (B) Box plots comparing the proportions of macrophage subsets between DL and ALD group. (C) Scatterplot showing the proportions of Macro_C1 and Macro_C3 across DL (*n* = 8), AH (*n* = 3) and AC (*n* = 19). (D) Dot plots showing significantly enriched GO terms in Macro_C1 and Macro_C3. (E) Violin plot comparing pathway scores of Macro_C1 and Macro_C3 from DL, AH and AC group. (F) UMAP plots of T/NK subsets. (G) Box plots comparing the proportions of T/NK subsets between DL and ALD group. (H) Box plots showing the proportions of CD4Tcm and MAIT cells across stages. (I) Violin plot comparing pathway scores of NK cells and indicated T cell subpopulations from DL, AH and AC group. *p* Values were calculated by two‐sided unpaired Wilcoxon test. *p < .05, **p < .01, ***p < .001,****p < .0001, ns, not significant.

To investigate the cellular state and function of immune cells in the liver tissues, we performed unsupervised clustering of myeloid cells and T/NK cells. Myeloid cells were clustered into 4 common lineages, monocytes, macrophages, dendritic cells and neutrophils based on canonical cell markers (Figure 6A). As macrophages obviously increased in both AH and AC samples (Figure S1B), we explored the heterogeneity and possible functions of macrophages in ALD. Composition analysis showed that Mac_C1 and C3 most significantly enriched in ALD compared with DL controls, especially in AC samples (Figure 6B and C). ECM modelling‐related genes, such as *FN1*, *SPP1* and *TGFβ* were highly expressed in Mac_C1 (Figure S7A), indicating desmoplastic features. Pathway analysis confirmed the enrichment of wound healing and extracellular matrix binding signals in Mac_C1 (Figure 6D). Immunofluorescent staining confirmed the increase of CD68^+^FN1^+^ macrophages in ALD livers (Figure S7B and C). Mac_C3 expressed high levels of *CX3CR1, TREM2, C3* and *CCL2* (Figure S7A), which have been reported as scar‐associated and pro‐inflammatory genes.^22^ In addition, pathway analysis revealed that lymphocyte mediated immunity and antigen processing signalling were enriched in Mac_C3 (Figure 6D). We further analysed gene expression and pathway enrichment of these subpopulations across ALD stages. Of note, we found that pathways related with activation, like phagocytosis, cytokine production and leukocyte cell adhesion, were mostly activated in the two macrophage populations from AH group, while downregulated in those from AC group (Figure 6E). In contrast, antigen processing and presentation pathways were exclusively upregulated in Mac_C1 and C3 from AC group (Figure 6E). These changes in macrophages might be an important reason for active inflammation responses in AH stage and immunosuppressive microenvironment in advanced AC stage.

The T/NK cells were classified into CD4^+^ T cells, CD8^+^ T cells, NKT cells, NK cells and γδT cells. CD4^+^ and CD8^+^ T cells were reclustered into naïve T cells; central memory CD4^+^ T subsets (CD4 Tcm); Treg, effector T; CD8 effector memory (CD8 Tem); tissue resident memory (Trm); IFN‐response; NK like; mucosal‐associated invariant T (MAIT) cells (Figure 6F). The percentages of CD4 Tcm increased significantly in ALD samples, and showed a progressive increase from DL control to AH to AC (Figure 6G and H). As noted by others,^23^, ^24^ we found that MAIT cells significantly decreased in ALD livers and exhibited a gradual manner (Figure 6G and H), implying potential roles in disease progression. Furthermore, we compared the gene expression and pathway activity across DL and ALD stages. We noticed that numerous cytokines and chemokines, such as *CCL3L1, CCL4L2, CXCL2, CXCL3, XCL1, CCL3* and *CCL5*, were downregulated in NK cells and CD4 Tcm from AC group compared to AH group (Tables S4 and S5). Meanwhile, essential genes for T/NK cell mediated cytotoxicity, such as *IFNG, TNF, CSF2, KLRD, KLRC2*, were also downregulated in these populations from AC group compared to those from DL and AH group (Tables S4 and S5). In accordance with the gene expression profiles, pathway analysis suggested that pathways related with NK/T cytotoxicity functions were dramatically upregulated in NK/T populations from AH group, such as NK, effector T cells, CD4 Tcm and MAIT cells, but uniformly decreased in AC group (Figure 6I), which indicating attenuated T/NK cell immune defence activities during the advanced ALD progression.

### 3.7 Characteristics of fibroblasts and endothelial cells

**FIGURE 7:**
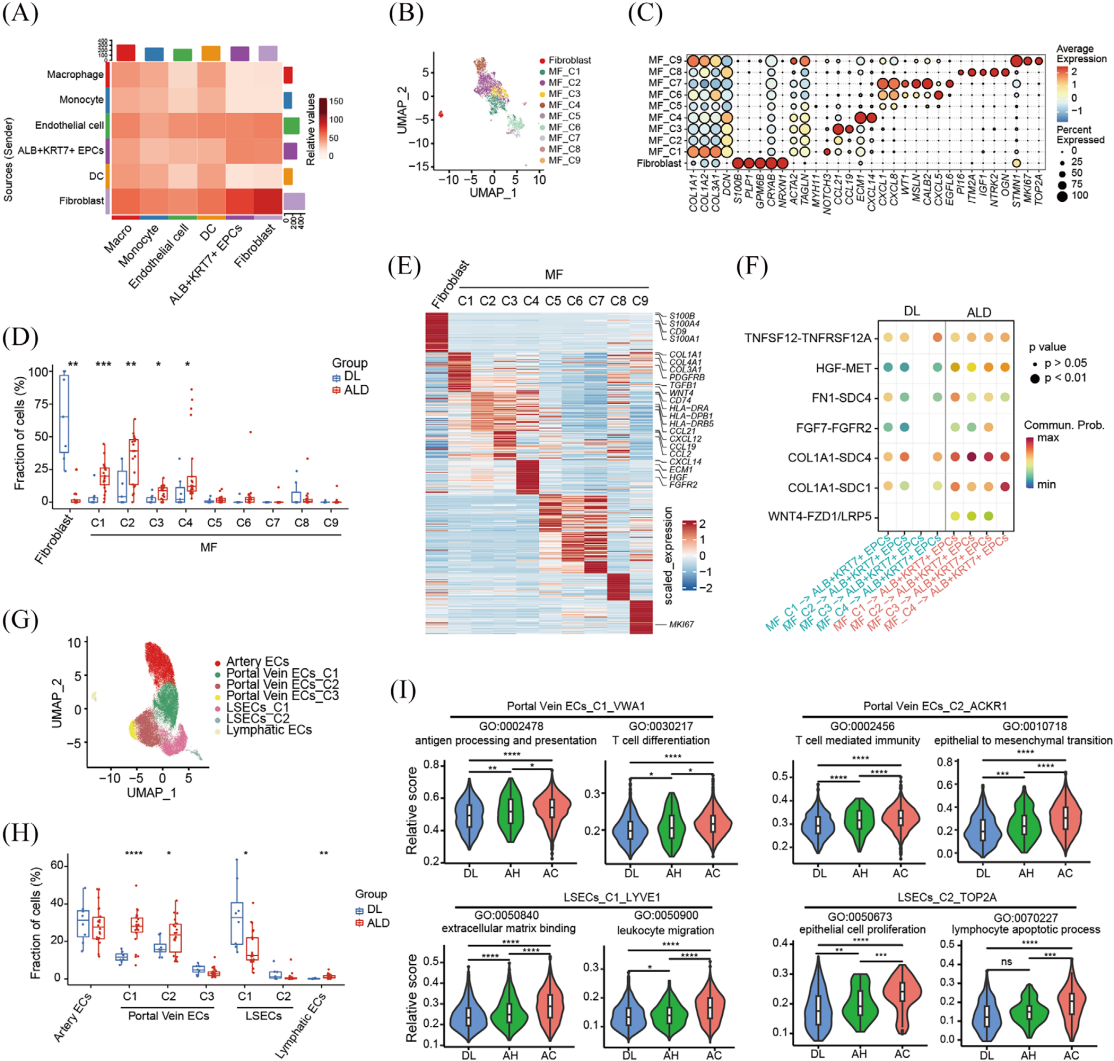
Characteristics of fibroblasts and endothelial cells (A) Heatmap showing the potential interaction intensity between indicated cell types. **(B)** UMAP plots of fibroblast subsets. **(C)** Bubble heatmap showing expression levels of marker genes across fibroblast subsets. **(D)** Box plots showing the proportions of fibroblast subsets in DL and ALD group. **(E)** Gene expression heatmap in fibroblast subsets. **(F)** Dot plot of ligand–receptor interactions between fibroblast subsets C1 to C4 and ALB^+^KRT7^+^ EPCs from DL and ALD group. **(G)** UMAP plots of endothelial cell subsets. **(H)** Box plots showing the proportions of endothelial cell subsets in DL and ALD group. **(I)** Violin plot comparing pathway scores of indicated endothelial populations from DL, AH and AC group. *p* Values in were calculated by two‐sided unpaired Wilcoxon test. *p < .05, **p < .01, ***p < .001,****p < .0001, ns, not significant.

ALD initiation and progression involved a complex interplay between multiple cell lineages. To identify potential interactions between the above analysed cell types, we used CellPhoneDB to perform an unbiased cell interaction analysis between these populations, including ALB^+^KRT7^+^ epithelial cells, fibroblasts, endothelial and immune cells. The results showed that fibroblasts and endothelial cells dominated the interactions with ALB^+^KRT7^+^ epithelial cells (Figure 7A). Then the liver fibroblasts were classified into 10 subgroups: S100B^+^ fibroblasts and 9 myofibroblast subsets (Figure 7B and C). The S100B^+^ fibroblasts were dominantly enriched in DL tissues, and remarkably decreased in ALD liver tissues (Figure 7D). While four clusters of myofibroblasts (MF_C1, MF_C2, MF_C3 and MF_C4) were preferentially expanded in ALD livers, in particular AC livers (Figure 7D). The DEG analysis showed that MF_C1 and MF_C4 expressed high levels of fibrillar collagens and pro‐fibrogenic genes, such as *COL1A1, COL1A2, COL3A1, ECM1* and *PDGFRB*, implying extracellular matrix producing and organisation capacities (Figure 7E). MF_C2 and MF_C3 expressed high levels of chemokines ligand, such as *CCL19, CCL21, CCL2, CXCL2, CXCL12*, indicating active crosstalk with other cells (Figure 7E). By analysing gene expression profiles across DL and ALD livers, we found that *S100A8* and *SELP* mildly increased in MF‐C1 from AH group, indicating potential immunoregulating functions of these cells during early ALD progression (Table S6). To further identify the key mediators for cell communications between ALB^+^KRT7^+^ EPCs and fibroblasts, especially MF‐C1 to C4 subsets that expanded in ALD livers, the ligand‐receptors were compared between DL and ALD group. In comparison to fibroblasts from DL livers, ALD fibroblasts exhibited strengthened crosstalk with ALB^+^KRT7^+^ EPCs via HGF‐MET and WNT4‐FZD/LRP5/6 (Figure 7F).

The endothelial cells were subclustered into 7 populations, including 1 artery ECs (*CXCL12^+^*), 3 Portal Vein ECs, 2 sinusoidal endothelial cells (LSECs) and lymphatic ECs (Figure 7G). Among these populations, the Portal Vein ECs_C1, C2 and Lymphatic ECs showed significant enrichment in ALD group (Figure 7H). LSECs located in the hepatic sinusoids, are the first to contact hepatic blood flow and are also the earliest cells to be damaged in liver diseases. Consistently, we observed significant decreased LSECs in ALD compared to DL livers, especially in AC samples (Figures 7H and S8A). The DEG analysis showed that Portal Vein ECs C1 expressed high levels of extracellular matrix organisation genes such as *COL15A1, COL4A2, PDGFD* and *PDGFB* (Figure S8B), defined as fibrogenic‐related EC. Portal Vein ECs_C2 expressed high levels of immune adhesion‐related genes, for example, *ACKR1, SELE, SELP*, and chemokines *CCL14, CXCL2* (Figure S8B), were defined as immune adhesion‐related EC. Similar endothelial populations (Portal Vein ECs C1 and C2) in cirrhotic livers have been annotated as scar‐associated endothelium.^22^ By analysing gene expressions of the EC populations across disease stages, we found that although the scar‐associated endothelial populations (Portal Vein ECs C1 and C2) showed no apparent cell abundance changes in the AH samples compared to DL livers, gene expression patterns have quietly changed. We observed mildly increased expressions of leukocyte cell recruitment or adhesion‐related genes, such as *S100A8, S100A9, HLA‐DQA1* and *HLA‐DRA* in Portal Vein ECs C1 and C2 from AH group (Tables S7 and S8). While, in the advanced AC stage, immunoregulating functions of these scar‐associated endothelial populations emerged more prominent, evidenced by extensively increased chemokines and immune adhesion molecules expression, such as *SELE, CCL2* and *ACKR1* (Tables S7 and S8). Moreover, pathway analysis suggested that antigen processing/presentation and T cell differentiation pathways were progressively upregulated in Portal Vein ECs_C1 populations across ALD stages (Figure 7I). Of note, EMT signalling gradually increased in Portal Vein ECs_C2 as ALD progression, implying possible roles in regulating epithelial cell transformation (Figure 7I). Pathways associated with ECM binding and immune cell migration gradually increased in LSECs_C1 from DL to AH and AC livers (Figure 7I). Intriguingly, we found that lymphocyte apoptotic signal exclusively dramatically upregulated in LSECs_C2 of AC samples (Figure 7I). These findings together suggested that endothelial cells might play vital immunoregulating functions during ALD progression.

## 4 DISCUSSION

Using scRNA‐seq, we measured the transcriptomes of all liver cell types obtained from patients with ALD as well as healthy donors. ALD is a metabolic disease triggered by alcohol‐mediated liver damage, so we first focused on liver parenchymal cells. In contrast to normal hepatocytes loss, novel subsets of hepatocytes that expressing progenitor and cholangiocyte markers aggressively expanded in ALD livers. As previously reported, hepatocytes will dedifferentiate into progenitor like cells to escape injury through a reversible process during chronic injury.^25^

Surprisingly, we identified a subset of epithelial cells that expressed both ALB and KRT7. The UMAP position of the ALB^+^KRT7^+^ epithelium was clearly separate from that of mature hepatocytes and biliary epithelial cells. In case of the of stem cell gene expression pattern, the ALB^+^KRT7^+^ epithelium exhibited strong similarity with biliary epithelial cells. Thus in several previous studies, researchers did not distinguished these progenitor‐like populations from biliary epithelial cells.^26^, ^27^ However, the cell cycle distribution of ALB^+^KRT7^+^ epithelium was very different from that of mature epithelial cells. It has been reported that in normal liver, only a very small number of liver cells (less than 1%) engage in DNA synthesis.^28^ While the proportion of S‐phase cells in ALB^+^KRT7^+^ epithelium is significantly high, reaching about 30%. Moreover, we also found that compared with mature epithelial cells, ALB^+^KRT7^+^ epithelium contained higher proportions of unspliced RNA, implying its active transcription status and in an early stage of cell evolution.^20^ Pseudo‐temporal cell evolution analysis proved that ALB^+^KRT7^+^ epithelium had bidirectional transformation potential. Taken together, we concluded that the ALB^+^KRT7^+^ epithelium is a distinct epithelial population.

Previous experimental investigations have demonstrated that such biphenotypic cells involved in the regenerative process in both acute injury and chronic liver disease.^29^, ^30^ Recent single‐cell RNA sequencing studies have also identified similar epithelium populations. For instance, Pu et al. identified a BEC originated CK19^+^HNF4α^+^ epithelium subset, which can differentiate into hepatocytes during severe liver injury, implying a liver regeneration function.^31^ Another single‐nucleus RNA sequencing study on patients with metabolic dysfunction‐associated steatotic liver disease (MASLD) also reported an intermediate epithelial population, co‐expressing cholangiocyte and hepatocyte markers, and demonstrated their involvement in liver regeneration.^32^ In our study, by investigating patients across different stage of ALD, we observed varied functions of ALB^+^KRT7^+^ epithelium. ALB^+^KRT7^+^ epithelium from AH samples were more related with liver repair, evidenced by enriched ECM and wound healing pathways. While in ALB^+^KRT7^+^ epithelium from AC samples, in addition to cell proliferation‐related pathways (e.g., MAPK signalling), tumour‐related pathways were significantly enriched, suggesting malignant transformation potential. Higher CNV scores of the ALB^+^KRT7^+^ population in AC group reinforced their malignant transformation potential. These together indicated that in early or short‐term injuries, the biphenotypic epithelium mainly contribute to liver repair, while in advanced ALD stage, the characteristic and function of such epithelium have changed.

It has been reported that adult human liver‐derived organoids were originated from biliary epithelial cells‐derived bipotent progenitor cells. These organoids expressed stem cell marker *PROM1* and *LGR5*, as well as ductal (*SOX9, OC2*) and hepatocyte markers (*HNF4α*).^33^ We assumed that the reported biliary duct derived EPCAM^+^ bipotent progenitor is the ALB^+^KRT7^+^ population in our study. This speculation was validated by positive expression of ALB, KRT7 and EPCAM in the liver organoids. Highly enriched cell division and DNA replication pathways in ALD organoids were strong consistent with cell proliferation status in single‐cell RNA analysis. In addition, tumourigenesis associated pathways (e.g., stem cell pathway, angiogenesis and fibroplasia) were dramatically enriched in ALD organoids. In accordance with the single‐cell results that Wnt signal enriched in ALB^+^KRT7^+^ population, the gene expressions of in vitro cultured ALD organoids also exhibited increased Wnt activity. Emerging evidence suggested that Wnt pathway not only play essential functions in tissue development and tumourigenesis, it also exert important roles in fibrotic responses and modulating Wnt pathway can be a potential strategy to reverse liver fibrosis.^34^, ^35^ A recent single‐cell RNA study on mouse model indicated that Wnt ligands: Wnt7a, Wnt7b, and Wnt10a were dramatically upregulated in biliary epithelial cells from DDC‐treated mice.^36^ Here, in our data, Wnt7a is the most significant upregulated Wnt ligand in ALD organoids, as well as its strong correlations with top ALB^+^KRT7^+^ population genes (*CXCL6* and *CCN1*) in AC livers underlined the pivotal role of Wnt7a in ALB^+^KRT7^+^ EPCs and ALD. Previous studies revealed that Wnt7a acts in a paracrine fashion through the canonical Wnt pathway by binding to receptor Frizzleds and activates β‐catenin signal in various tumours.^37^, ^38^ In line with these findings, our data suggested that ALD organoids that highly expressed Wnt7a exhibited significant tumour‐promoting effect through activating β‐catenin in vitro and in vivo. Blockade Wnt protein secretion or knockdown Wnt receptor‐FZD5 in HCC cells reversed the tumour‐promoting effect of ALD organoids. These data together indicated that the ALB^+^KRT7^+^ population in advanced ALD livers not only possess malignant transformation potential, but also have adverse tumour‐promoting effect. Alcohol metabolites will inevitably induce accumulation of gene mutations in liver cells, once ALB^+^KRT7^+^ population transform to malignant cells due to gene mutation or other malignant cells appear, the existing ALB^+^KRT7^+^ populations promote the tumour initiation and progression. Nevertheless, additional studies, for example, by deleting the Wnt ligands in ALB^+^KRT7^+^ population or organoids, are necessary to demonstrate the exact role of Wnt7a in the malignant progression of ALD. Interestingly, we noticed that ALB^+^KRT7^+^ population highly expressed *CCN1* (also called *CYR61*), a YAP target gene, indicating increased YAP activity in the ALB^+^KRT7^+^ population. Previous study showed that YAP signalling is required for progenitor‐like cell survival under injury context.^36^ Additionally, Wnt/β‐catenin has been revealed to crosstalk with YAP in intrahepatic BECs, which are required for the carcinogenesis in mouse liver tissues.^39^ In this context, further investigations are needed to address the requirement of the YAP signalling in ALB^+^KRT7^+^ epithelium expansion and Wnt activation during the ALD progression.

The analysis of immune cells, including macrophages, T and NK cells, demonstrated increased immune activities in AH stage, but suppressed state in AC stage. Coherently, we observed more immunosuppressive‐like tumour environment in alcohol‐related HCC with high ALB^+^KRT7^+^ population abundance. In addition, ALB^+^KRT7^+^ populations expressed higher chemokines than other epithelial cells. These collectively implicated possible immunomodulating roles of ALB^+^KRT7^+^ EPCs.

We observed that nonparenchymal populations associated with fibrogenesis similar to previous reports,^22^ including fibrogenic fibroblasts (highly express type I collagen and type III collagen) and scar‐associated endothelial cells (marked by *PLVAP* and *ACKR1*). Through the cell‐cell communications analysis, we found possible mechanism clues that may lead to ALB^+^KRT7^+^ EPCs expansion and transformation, such as fibroblasts derived HGF and WNT4. Fibroblast‐derived HGF has been revealed to promote liver progenitor cell expansion through HGF‐MET signal.^40^, ^41^ WNT4, a member of Wnt family, has been reported to promote EMT in colorectal cancer cells.^42^ Additionally, liver endothelial cells, adjacent to both epithelial cells and immune cells, have been demonstrated as communication hubs that interact with all other liver cell types.^43^ Here, we also observed strong interactions between endothelial cells and ALB^+^KRT7^+^ EPCs, together with increased epithelial proliferation and EMT‐related pathways in endothelial subpopulations from AC group, we supposed potential epithelial‐regulating functions of the endothelial populations. Further experiments are needed to investigate the detailed mechanisms

There are also limitations in our study. Firstly, due to the liver cells, in particular the epithelial cells are easily crushed during the tissue dissociation process, only relative small ratio of epithelial cells were obtained. Future studies using single‐nuclear RNA sequencing are needed to further validate the epithelial findings. Moreover, as it is impractical to get large number of liver samples for early alcohol‐related hepatitis or fibrosis (AH) patients, limited sample size might potentially lead to reduced statistical power and increased risk of bias in the analysis of early ALD. In the following study, more liver tissue samples collections are needed to validate the above findings in AH patients. Additionally, the existing bulk RNA‐seq data from public database could also be assessed using gene deconvolution model to validate changes in cell‐type proportions.

In summary, using sc‐RNA, we deciphered the components and phenotype changes of liver cells across different human ALD stages. Particularly, we identified the biphenotypic ALB^+^KRT7^+^ epithelium enriched in ALD livers and exhibit varied functions across ALD stages. ALB^+^KRT7^+^ epithelium from advanced ALD had malignant transformation potential and tumour promotion activity. Our study provides important insights that Wnt targeted therapy can prevent malignant progression for patients with advanced alcohol‐related cirrhosis.

## AUTHOR CONTRIBUTIONS

Hongyang Wang and Jing Fu conceived and designed the experiments; Shuijun Zhang provided human samples; Xiaofang Zhao, Senyan Wang, and Qi Liu performed the experiments and bioinformatics analysis; Wenjuan Wei and Xiaoyan Sun analysed the data; Hao Song and Jing Xu collected the human specimens; Jing Fu, Xiaofang Zhao, and Senyan Wang wrote the manuscript. All authors have read, revised, and approved the final manuscript.

## CONFLICT OF INTEREST STATEMENT

The authors declare no competing interests.

## Supporting information



Supporting Information

Supporting Information

Supporting Information

Supporting Information

Supporting Information

Supporting Information

Supporting Information

Supporting Information

Supporting Information

Supporting Information

## Data Availability

Data are available upon reasonable request. All data relevant to the study are included in the article or uploaded as Supplementary Information. The raw data for single‐cell RNA sequencing reported in this publication can be accessed under the Genome Sequence Archive (https://ngdc.cncb.ac.cn, accession number HRA009931) on request.
