# Supplementary material for: Single‐cell landscape of the intrahepatic ecosystem in alcohol‐related liver disease
Source: Clin Transl Med. 2025 Jan 20;15(1):e70198. doi: 10.1002/ctm2.70198 (PMC11746962; doi:10.1002/ctm2.70198)
Supplement: Supplementary file 1 — Supporting Information [file CTM2-15-e70198-s010.pdf]

Supplementary Figures

Supplementary Figure1

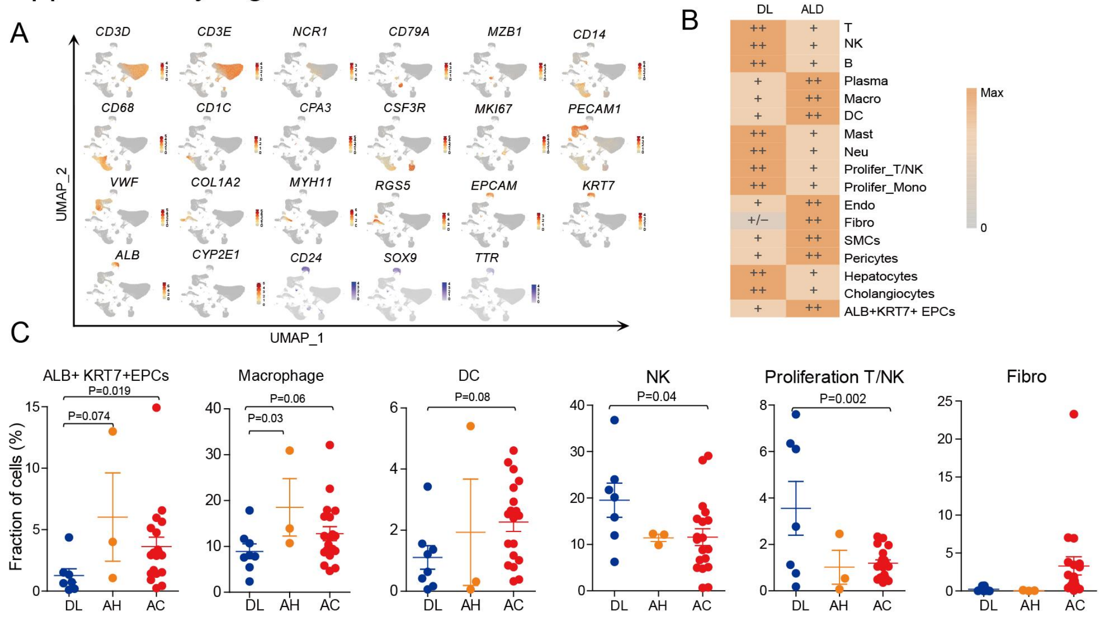

Supplementary Figure1: **A**.UMAP plots showing expression levels of marker genes. **B**. Prevalence of each cell type estimated by Ro/e score (++, 1 < Ro/e ⩽ 3; +, 0.2 ⩽ Ro/e ⩽ 1; +/−, 0 < Ro/e < 0.2). **C.** Scatterplot showing the proportions of indicated cell types across normal livers (DL, n=8), alcohol-related hepatitis/fibrosis (AH, n=3) and alcohol-related cirrhosis livers (AC, n=19). Wilcoxon rank-sum test was used for statistical analysis.

Supplementary Figure2

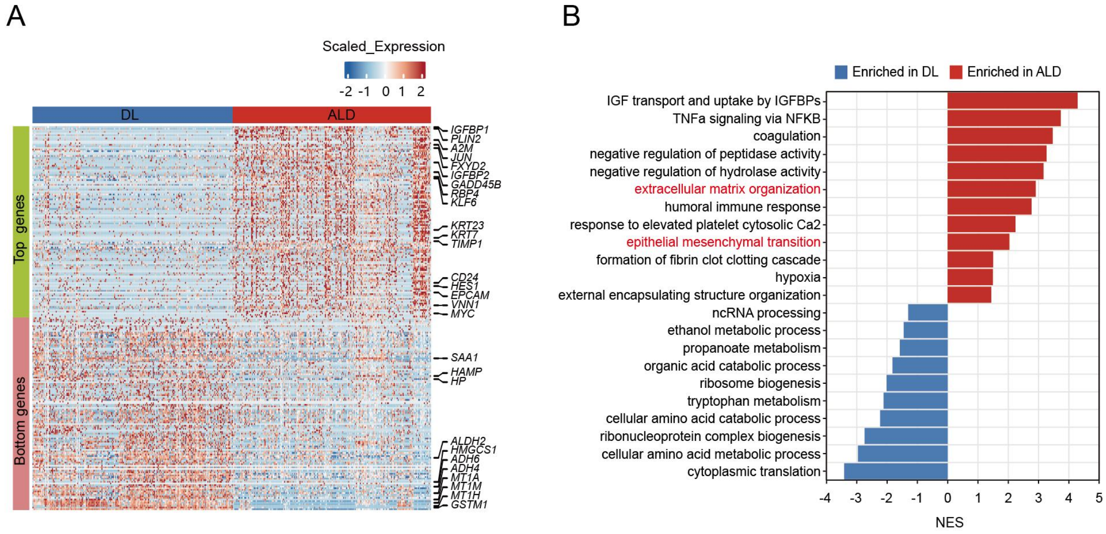

Supplementary Figure2: **A**. Heatmap showing top-ranking DEGs between hepatocytes from DL and ALD group. **B**. Bar chart showing the highest differential pathways between hepatocytes from DL and ALD samples, based on GSEA. Red bars represent pathways that were enriched in ALD hepatocytes, and blue bars represent pathways that were enriched in DL hepatocytes.

Supplementary Figure3

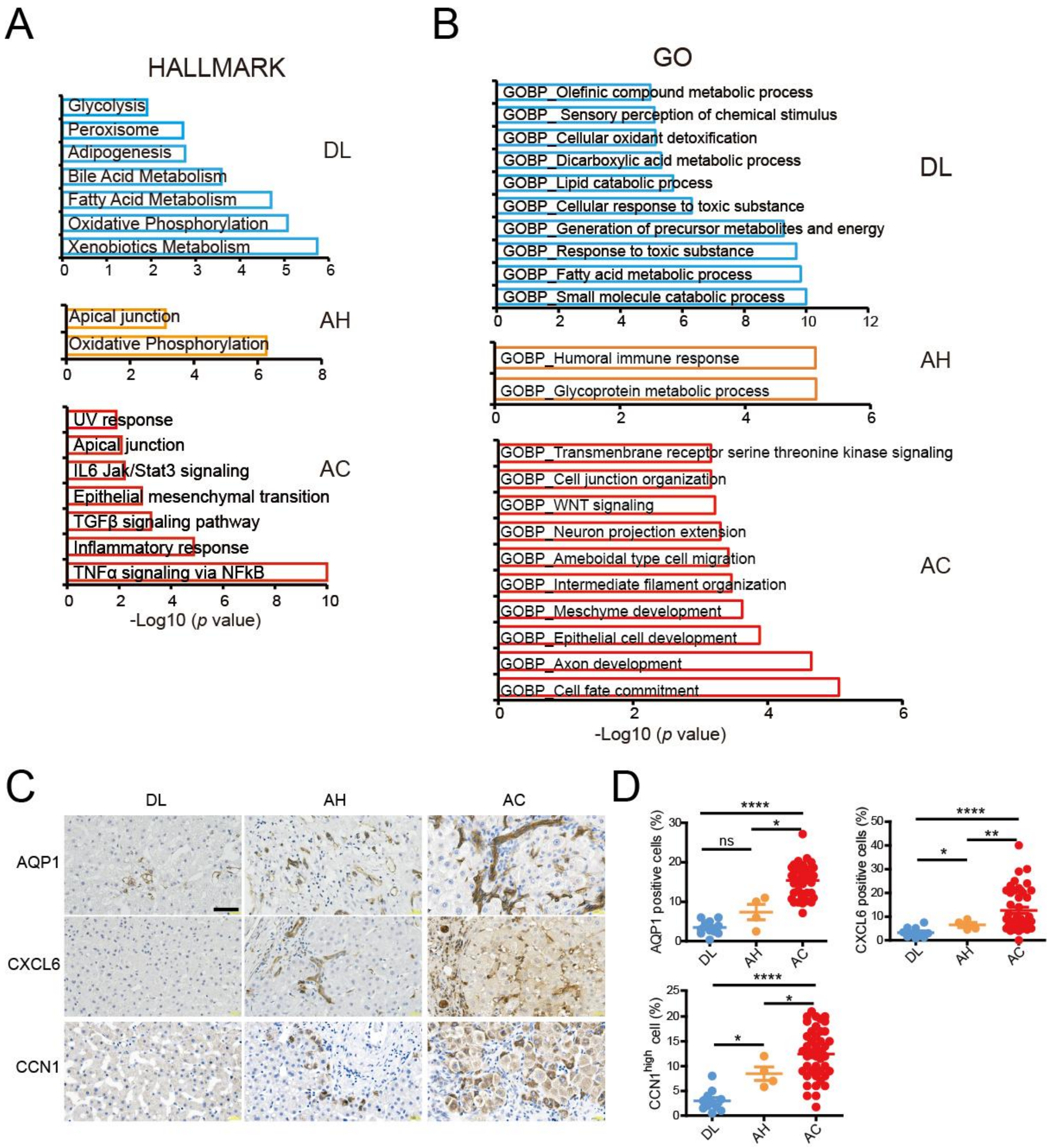

Supplementary Figure3: **A.** Bar chart showing the enrichment of HALLMARK pathways in ALB^{+}KRT7^{+} EPC from DL, AH and AC groups, based on GSEA pathway analysis. **B.** Bar chart showing the enrichment of GO pathways in ALB^{+}KRT7^{+} EPC from each group. **C.** Representative IHC staining images of AQP1, CXCL6 and CCN1 in DL, AH and AC samples. Scale bars, 50μm. **D.** Boxplot illustrating the fraction of positive cells, in samples from each group based on IHC results. *P < 0.05; **P < 0.01; ****P < 0.0001; ns, not significant. Wilcoxon rank-sum test was used for statistical analysis.

Supplementary Figure4

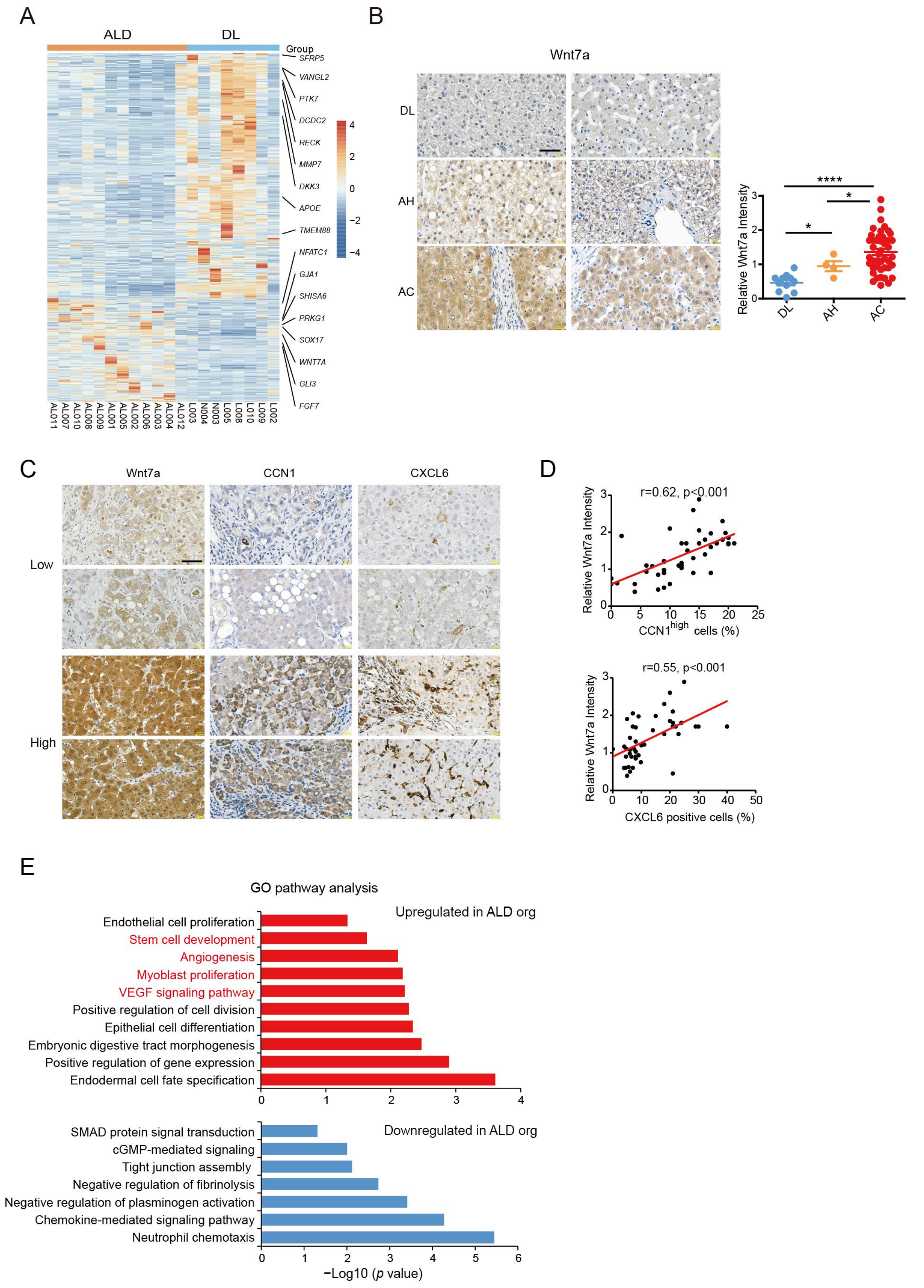

Supplementary Figure4: **A**. Heatmap showing top-ranking DEGs for organoids of DL and ALD groups. **B**. Representative IHC staining images of Wnt7a in DL, AH and AC liver sections (left). Scale bars, 50μm. Boxplot comparing the relative intensities in each group based on IHC results. Wilcoxon rank-sum test was used for statistical

analysis. **C**. Representative IHC staining images of low- and high-expression levels of Wnt7a, CCN1 and CXCL6 in AC livers. Scale bars, 50μm. **D.** Correlation plot of the Wnt7a expression levels and $CCN1^{high}$ or $CXCL6^{+}$ cell fractions in 45 AC livers. Pearson correlation was performed for statistical analysis. **E.** Bar chart showing the enrichment of specific pathways in ALD organoids compared with DL group, based on GO pathway analysis.

Supplementary Figure5

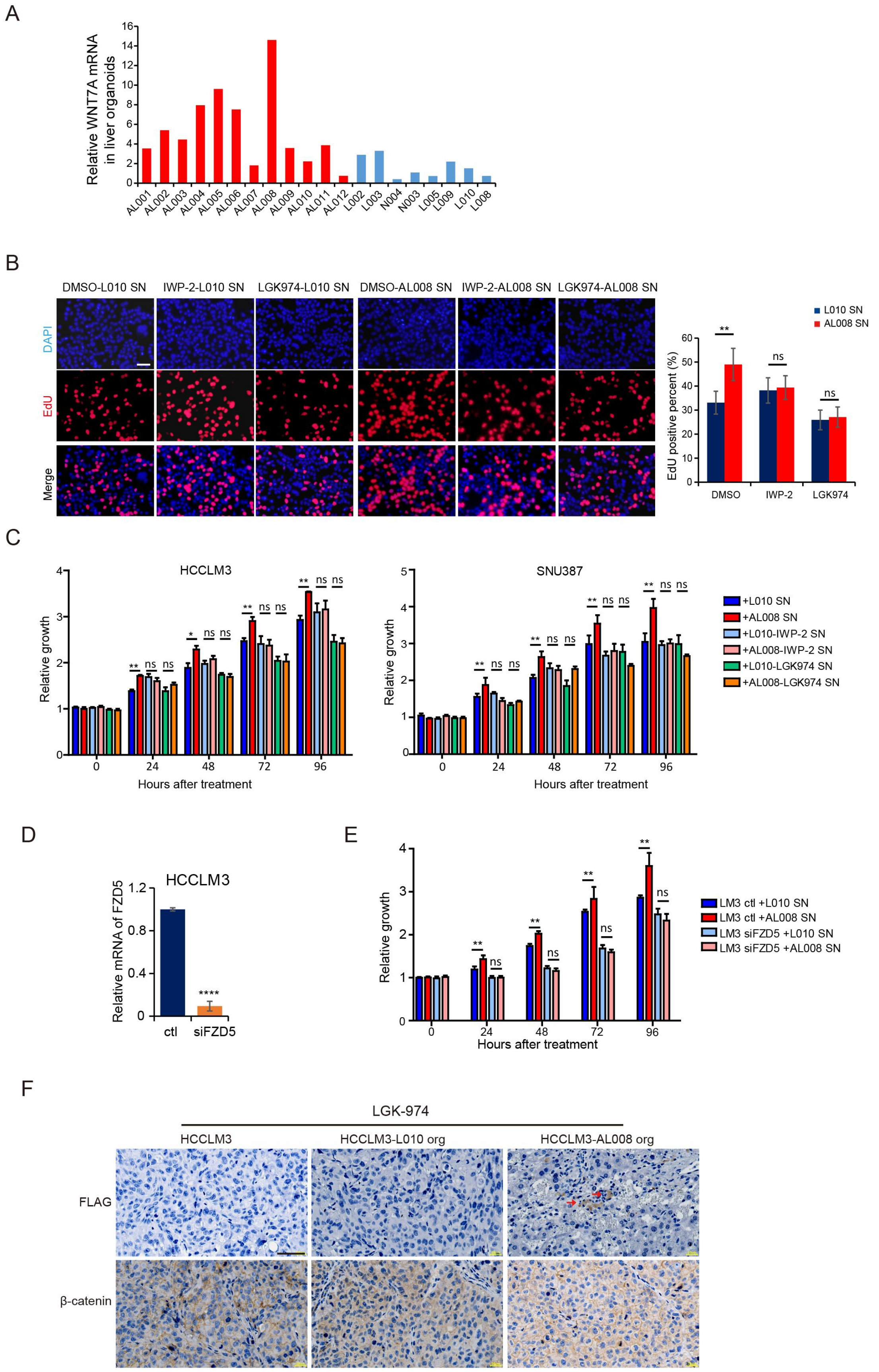

Supplementary Figure5: **A**.Relative WNT7A mRNA levels in each liver organoid

derived from DL and ALD livers, based on bulk RNA-seq data. **B**. L010 and AL008 organoids were treated with IWP-2 or LGK974, or DMSO as vehicle control, after 48 hours, supernatants were collected for HCCLM3 cell culture. Representative EdU staining images of HCCLM3 cells treated with L010 SN or AL008 SN for 48 hours. Scale bars, 50 μm. Boxplot illustrating the fraction of EdU positive cells. **C.** Boxplot showing the cell growth ratios of HCCLM3 and SNU387 cells in different group, detected by CCK8 assay. **D**. Quantitative PCR analysis of FZD5 expression levels in HCCLM3 cells transfected with siFZD5 or control lentiviral vectors. **E.** Cell growth of HCCLM3 in different group was detected by CCK-8 assay. **F.** Representative IHC staining images of FLAG and β-catenin in in tumor sections of each group. Scale bars, 50 μm. Wilcoxon rank-sum test was used for statistical analysis.

Supplementary Figure6

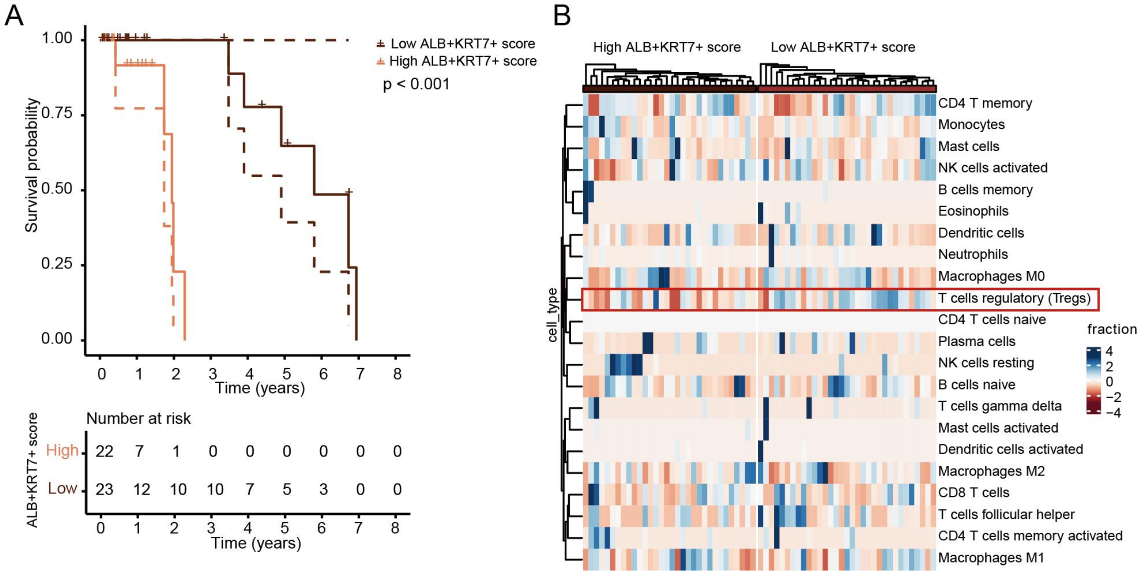

Supplementary Figure6: **A.** Analysis of the correlation between ALB$^+$KRT7$^+$ EPC scores and overall survival prognosis in 45 alcohol-related HCC samples from TCGA-LIHC corhort. **B**. The 65 alcohol-related HCC samples from TCGA-LIHC corhort were devided into high- and low-ALB$^+$KRT7$^+$ EPC group. Heatmap showing the immune cell infiltration levels in different group.

Supplementary Figure7

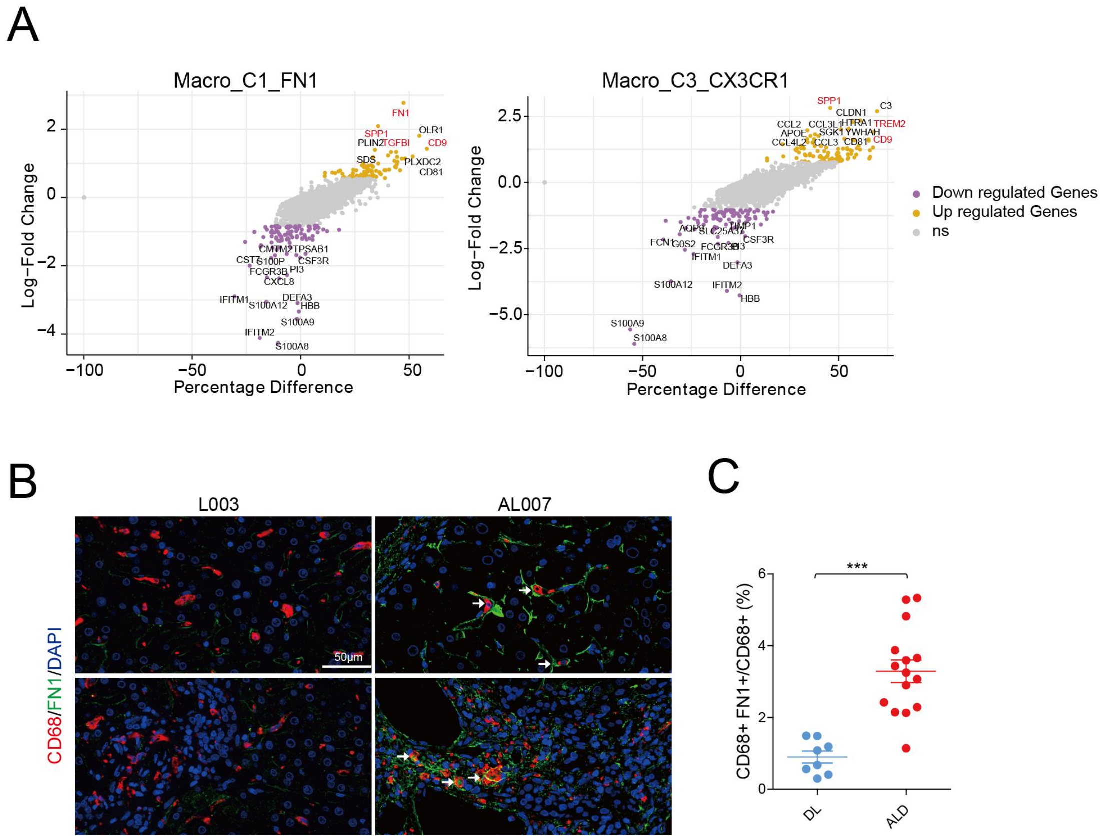

Supplementary Figure7: **A.** Volcano plot showing highly differentially expressed genes in Macro_C1_FN1 and Macro_C3_CX3CR1. Yellow dots represent significantly up-regulated genes (log2FC >1, adjusted P<0.05); purple dots represent significantly down-regulated genes (log2FC<-1, adjusted P<0.05); and gray dots represent genes with no significant difference. **B**. Representative immunofluorescence images of CD68 and FN1 in donor control and alcohol-related cirrhosis liver sections. White arrows indicate the distribution of $CD68^{+}$ $FN1^{+}$ macrophages. Scale bar, 50 μm. **C.** Boxplot comparing the ratio of $CD68^{+}FN1^{+}$ macrophages relative to $CD68^{+}$ macrophages between DL (n=8) and ALD (n=15) samples. ***P < 0.001. Two-tailed paired Student's t-test was used for statistical analysis.

Supplementary Figure8

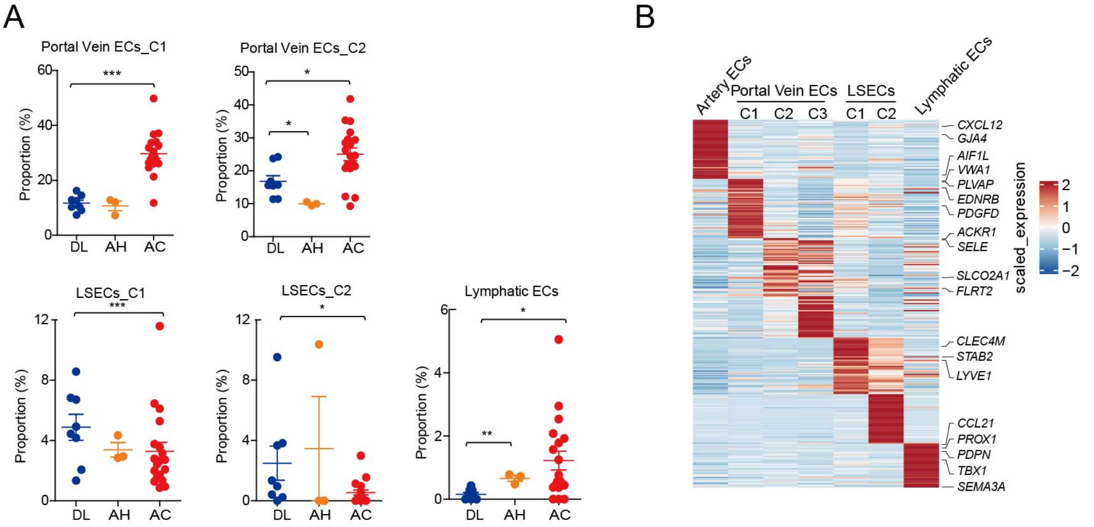

Supplementary Figure8: **A**. Scatterplot comparing the frequencies of endothelial cell subtypes across donor livers (DL, n=8), alcohol-related hepatitis/fibrosis (AH, n=3) and alcohol-related cirrhosis livers (AC, n=19). *P < 0.05; ***P < 0.001. Wilcoxon rank-sum test was used for statistical analysis. **B**. Gene expression heatmap of endothelial cell subsets.
