# Supplementary material for: Single‐cell landscape of the intrahepatic ecosystem in alcohol‐related liver disease
Source: Clin Transl Med. 2025 Jan 20;15(1):e70198. doi: 10.1002/ctm2.70198 (PMC11746962; doi:10.1002/ctm2.70198)
Supplement: Supplementary file 2 — Supporting Information [file CTM2-15-e70198-s005.docx]

**Supplementary Methods**

***Single cell collection, sorting, library preparation and sequencing***

Fresh liver tissue samples were surgically removed from patients and immersed in tissue storage solution (Miltenyi) and transported to the lab in a refrigerated container.

Liver tissues were cut into approximately 1-2 mm^3^ pieces in the RPMI-1640 medium (GIBCO) with 10% fetal bovine serum (FBS, GIBCO), and enzymatically digested with gentleMACS (Miltenyi) for 60 min on a rotor at 37°C according to manufacture’s instruction. The dissociated cells were subsequently passed through a 100 µm SmartStrainer and centrifuged at 400 g for 5 min. After the supernatant was removed, the pelleted cells were suspended in red blood cell lysis buffer (TIANDZ) and incubated on ice for 1-2 min to lyse red blood cells. After washing twice with 1×PBS (GIBCO), the cell pellets were re-suspended in sorting buffer (PBS supplemented with 1% FBS). The viability of cells would be checked on Countess® II Automated Cell Counter (Thermo Fisher) after trypan blue staining (Thermo Fisher).

The concentration of single cell suspensions was adjusted to 500-1200 cells/ul. Cells were loaded between 7,000 and 15,000 cells/chip position using the 10x Chromium Single cell 5’ Library, Gel Bead & Multiplex Kit and Chip Kit (10x Genomics, V3 barcoding chemistry) according to the manufacturer’s instructions. All the subsequent steps were performed following the standard manufacturer’s protocols. Purified libraries were analyzed by an Illumina Hiseq X Ten sequencer with 150-bp paired-end reads.

***Sequencing data processing***

Raw gene expression matrices were generated for each sample by the Cell Ranger (Version 6.1.2) Pipeline coupled with human reference version GRCh38-2020-A. The output filtered gene expression matrices were analyzed by R software (Version 4.1.2) with the Seurat package (Version 4.1.1). The percentage of mitochondrial transcripts for each cell (percent.mt) was calculated and added as metadata to the Seurat object. The Scrublet (Version 0.2.1) was used to remove the potential doublets from the datasets. Low quality cells were filtered before dimensionality reduction (n_genes>200, n_counts>500, percent_mito<10%). Expression values were then scaled to 10,000 transcripts per cells and Log-transformed. Effects of variable (percent.mt) was estimated and regressed out (ScaleData function, model.use=linear), and the scaled and centered residuals were used for dimensionality reduction and clustering.

***Dimensionality reduction and cell clustering***

We used 2,000 genes with high cell-to-cell variation, which were calculated using the FindVariableFeatures function in Seurat for further dimensionality reduction. To reduce dimensionality of each cell, the RunPCA function was conducted with default parameters on linear-transformation scaled data generated by the ScaleData function. Next, the ElbowPlot function was used to identify proper dimensions of each dataset. After projection of all cells into two-dimensional space by RunUMAP function, we initially built a graph of cells by using the K-Nearest Neighbors (KNN) algorithm. Classification of each cell type was inferred from the cluster-specific genes. Doublets were identified by searching for cells with substantial and coherent expression profiles from two or more cell types.

***Reclustering of major cell types***

To identify subtypes in different states, we used a two round clustering strategy. Firstly, cells belong to one major cell type were extracted from the count matrix. Then, dimensionality reduction, batch effect correction using harmony (Version 0.1.1) and cell clustering were performed.

***Differential expression genes (DEGs) identification and functional enrichment***

Differential gene expression testing was performed using the FindMarkers function in Seurat with parameter "test.use=t". Enrichment analysis for the functions of the DEGs was conducted using clusterProfiler (Version 4.7.1).

***Tissue distribution of clusters***

We calculated the Ro/e for each cluster in different tissues to quantify the tissue preference of each cluster. The expected cell numbers for each combination of cell clusters and tissues were obtained from the chi-squared test. One cluster was identified as being enriched in a specific tissue if Ro/e > 1. For most clusters, we used the Ro/e index (+++, Ro/e > 3; ++, 1 < Ro/e ≤ 3; +, 0.2 ≤ Ro/e ≤ 1; +/−, 0 < Ro/e < 0.2; and −, Ro/e = 0) to define the cluster preference in a specific tissue.

***Single-cell trajectory analysis***

Monocle (Version 2.22.0) aims to resolve cellular transitions during differentiation through pseudotemporal profiling of scRNA-seq data. After inputting the count matrix into the “newCellDataSet” function with its clustering information, it was computed into a lower dimensional space based on the discriminative dimensionality reduction with trees (DDRTree) method using 2000 high variation genes by FindVariable Features function.

Velocyto (Version 0.17.17) was run on the cellranger counts output folder to generate a loom file containing the spliced/unspliced counts matrix. The loom file was merged to an annadata object. The spliced/unspliced counts were then normalized using the package scVelo (Version 0.2.5) and genes without a shared splice count of 20 were filtered out of the annadata object. The moments were then computed across 40 neighbors and 2 PCs. Velocities were estimated using scVelo’s dynamical model, and velocities were then calculated and embedded onto monocle2’s dimensionality reduction result.

***Cell-cell communication analysis***

CellChat (Version 1.5.0) was used to comprehensively assess the communications among cells and quantitatively analyze intercellular communication networks. Briefly, the normalized data were loaded into CellChat and CellChatDB.human database was used to assess cell-cell communication in our dataset.

***SCENIC analysis***

The python package pySCENIC (Version 0.11.2) was used to run SCENIC analysis. The motifs database for Homo sapiens was downloaded from the website <https://pyscenic.readthedocs.io/en/latest/>. The input matrix was the normalized expression matrix of interesting cells.

***Gene set scoring***

AddModuleScore was used to calculate the scores of gene sets on individual cells.
